# Changes in tissue protein *N*-glycosylation and associated molecular signature occur in the human Parkinsonian brain in a region-specific manner

**DOI:** 10.1093/pnasnexus/pgad439

**Published:** 2023-12-25

**Authors:** Ana Lúcia Rebelo, Richard R Drake, Martina Marchetti-Deschmann, Radka Saldova, Abhay Pandit

**Affiliations:** CÚRAM, SFI Research Centre for Medical Devices, University of Galway, H91 TK33, Galway, Ireland; Department of Cell and Molecular Pharmacology and Experimental Therapeutics, Medical University of South Carolina, SC 29425, Charleston, USA; Institute of Chemical Technologies and Analytics, Vienna University of Technology, 1060 Vienna, Austria; CÚRAM, SFI Research Centre for Medical Devices, University of Galway, H91 TK33, Galway, Ireland; National Institute for Bioprocessing Research and Training (NIBRT), University College Dublin, A94 X099, Dublin, Ireland; School of Medicine, College of Health and Agricultural Science, University College Dublin, D04 V1W8, Dublin, Ireland; CÚRAM, SFI Research Centre for Medical Devices, University of Galway, H91 TK33, Galway, Ireland

**Keywords:** Parkinson's disease, human brain, *N*-glycosylation, protein glycosylation, glycomics, Biological Sciences, Neuroscience

## Abstract

Parkinson's disease (PD) associated state of neuroinflammation due to the aggregation of aberrant proteins is widely reported. One type of post-translational modification involved in protein stability is glycosylation. Here, we aimed to characterize the human Parkinsonian nigro-striatal *N*-glycome, and related transcriptome/proteome, and its correlation with endoplasmic reticulum (ER) stress and unfolded protein response (UPR), providing a comprehensive characterization of the PD molecular signature. Significant changes were seen upon a PD: a 3% increase in sialylation and 5% increase in fucosylation in both regions, and a 2% increase in oligomannosylated *N*-glycans in the substantia nigra. In the latter, a decrease in the mRNA expression of sialidases and an upregulation in the UPR pathway were also seen. To show the correlation between these, we also describe a small in vitro study where changes in specific glycosylation trait enzymes (inhibition of sialyltransferases) led to impairments in cell mitochondrial activity, changes in glyco-profile, and upregulation in UPR pathways. This complete characterization of the human nigro-striatal *N*-glycome provides an insight into the glycomic profile of PD through a transversal approach while combining the other PD “omics” pieces, which can potentially assist in the development of glyco-focused therapeutics.

**Significance Statement**

Parkinson's disease (PD) affects millions of people worldwide, but its complete molecular signature remains to be elucidated. Untangling PD-related molecular phenomena at the glycome level has been challenging due to the complexity of glycosylation and the lack of analytical tools. Here we characterize the nigro-striatal *N*-glycome in health and upon PD in human samples, and also the expression of glycosylation enzymes and the modulation of endoplasmic reticulum stress. Changes in the glycomic profile suggest a decrease in *N*-glycan complexity with disease progression, which also relates to the glyco-enzymes expressed. This study clarifies the human brain nigro-striatal *N*-glycome and identifies critical targets in the molecular signature of PD, which can be a reference for the future study of the brain *N*-glycome upon neurodegeneration.

## Introduction

Parkinson's disease (PD) is characterized by the death of dopaminergic neurons in the substantia nigra pars compacta due to the presence of Lewy bodies composed of protein aggregates, leading to neurodegeneration (1). Despite the increased knowledge and pathway-oriented research regarding PD pathophysiology, the study of how *N-*glycosylation in the brain correlates with the progression of the disease remains overlooked. In fact, very few studies have looked into the role of *N*-glycosylation in PD. So far this was only carried out in serum from patients, revealing a decrease in sialylation and an increase in fucosylation on tri-antennary glycans with two and three terminal sialic acids (2), and on IgG alone, showing that sialylation and biantennary galactosylated structures were also reduced in the PD group, as well as oligomannosylated structures (3). However, it is worth noting that *N*-glycosylation in PD brain tissue has so far been overlooked.

Glycosylation is a major highly regulated post-translation modification of proteins, modulating the protein's function, structure, and conformational stability (4). Since PD is associated with aberrant aggregation of α-synuclein, and glycosylation affects proteins’ structure and function, studying this feature is highly attractive. *N*-linked glycans are the most common type of glycans in eukaryotic glycoproteins since around 90% of these glycoproteins carry them (5). *N*-glycans can be categorized into three groups: oligomannose (only mannose residues are linked to the core structure); complex (branched structures are attached to the core); and hybrid (only mannose residues are linked to the man-α-[1,6] arm of the core and one/two branches starting with a GlcNAc residue on the man-α-[1,3] arm) (6). Their ubiquity and implication in every biological process highlight their importance. In the central nervous system (CNS) they play essential roles in differentiation, synaptogenesis, neurite outgrowth, and myelinogenesis during development (7). Congenital disorders of glycosylation (CDGs) are associated with different neuropathological symptoms such as seizures and stroke-like episodes (8), emphasizing how glycan dysregulations can affect the CNS.

*N*-glycosylation takes place within the endoplasmic reticulum (ER) and the Golgi apparatus, involving various glycosidases and glycosyltransferases that participate in the formation and trimming of the different glycosidic chains (9, 10) and chaperones that promote proper folding of the proteins. At the same time, a strict quality control system occurs by the glucosyltransferase uridine diphosphate glucose (UGGT) in the ER. This recognizes any misfolded glycoproteins (which could be due to aberrant *N*-glycosylation) and either promotes their re-glycosylation until proper folding or targets them for degradation (11–13). It is also in the ER that the first step in the assembly of *N*-glycans takes place, where the newly formed glycan chain is transferred from a lipid-linked oligosaccharide to the asparaginyl residues of the new glycoprotein's precursors, which is catalyzed by an oligosaccharyltransferase (4). The presence of metabolic deficiencies affecting this process such as tunicamycin administration has been described to be compensated by ER stress responses (14, 15). To decrease ER stress and inhibit the accumulation of misfolded proteins, the unfolded protein response (UPR) takes place, being activated through three different signal transducers: protein kinase-like ER kinase (PERK), protein kinase inositol requiring kinase 1 (IRE1) and activation transcription factor 6 (ATF6) (16). On the other hand, an insufficient ER stress response has been correlated with an increased susceptibility of mouse cerebellar neurons to *N*-glycosylation defects (17), emphasizing the bidirectional relationship between *N*-glycosylation and ER homeostasis (18). Thus, there are multiple players whose actions must be taken into account when characterizing the healthy vs. diseased “glyco”-environment.

**Fig. 1.**
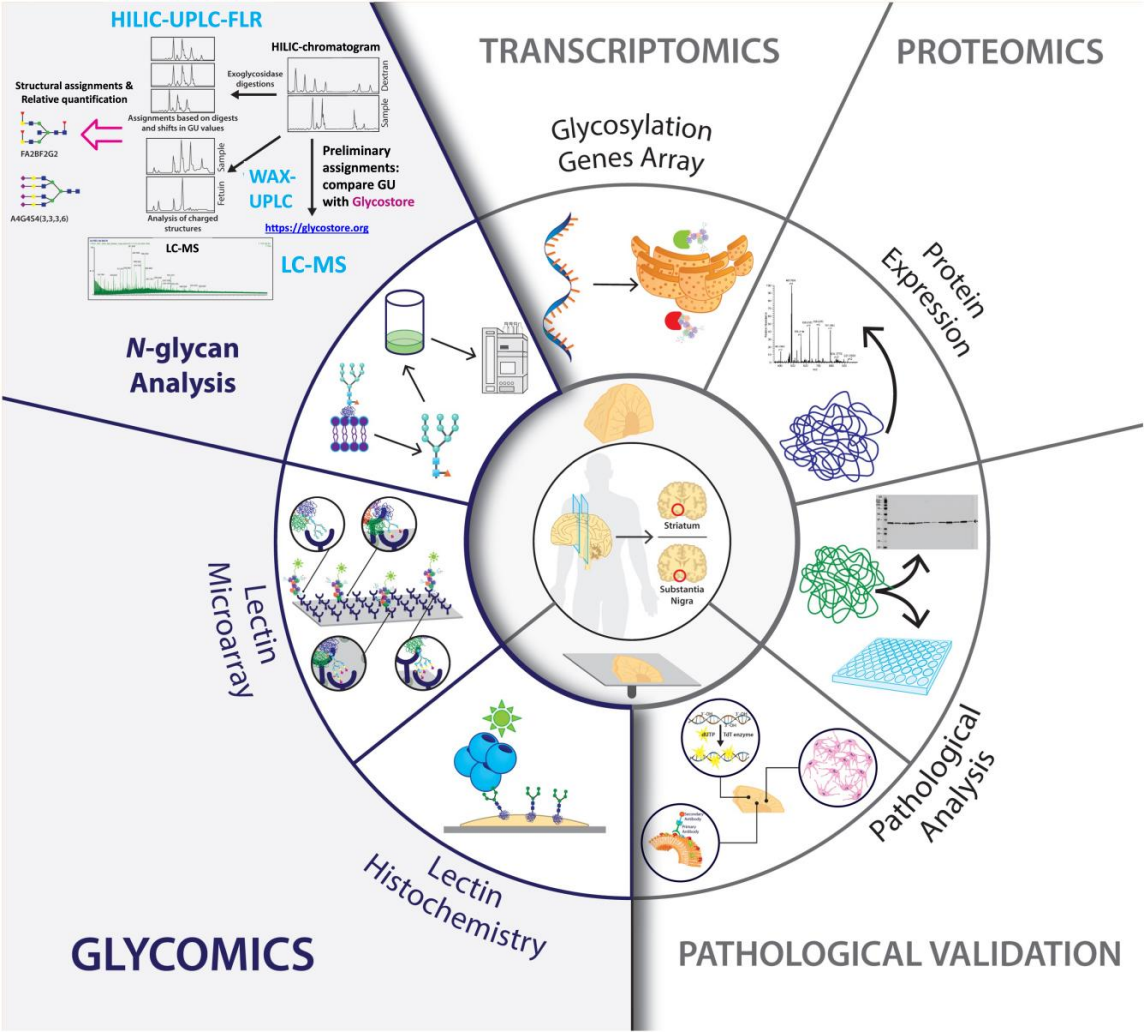
Schematic representation of the experimental design and procedures followed in this study. The study was designed for the region-specific and temporal characterization of the molecular signature in the Parkinsonian brain, with a focus on *N-*glycosylation. Two regions were analyzed (striatum and substantia nigra) from healthy subjects (*n* = 18), incidental Lewy-body disease (ILBD) patients (*n* = 3), and stages 3–4 PD patients (*n* = 15). Brain tissue from these patients was acquired either snap-frozen or in fixed-frozen sections. For the *N*-glycome studies, a multifaceted approach was developed using hydrophilic interaction ultraperformance liquid chromatography (HILIC–UPLC–FLR), exoglycosidase digestions, weak anion exchange liquid chromatography (WAX–UPLC–FLR), and LC–MS. Snap-frozen tissue was used to perform glycomic, transcriptomic, and proteomic analyses. Sections were used to validate the previous findings through matrix-assisted laser desorption/ionization MALDI-MSI (MALDI), and fluorescent and chromogenic histochemistry stainings.

This study represents a comprehensive spatio-temporal analysis of the human nigro-striatal protein *N-“*glyco” profile to understand how it is modulated along with other molecular players in PD (Fig. 1). Since glycosaminoglycans (GAGs) and *O*-glycome have recently been characterized in PD (19, 20), we explore the missing puzzle pieces to complete the characterization of protein glycosylation upon the onset of PD. This overview of the PD “-omics” is relevant since it allows for a more encompassing investigation of the modulation of *N*-glycosylation upon disease and how it interacts transversely with the overall molecular signature in the brain.

## Results

### Specific glycosylation trait changes are seen in the overall glycome upon PD progression

**Fig. 2.**
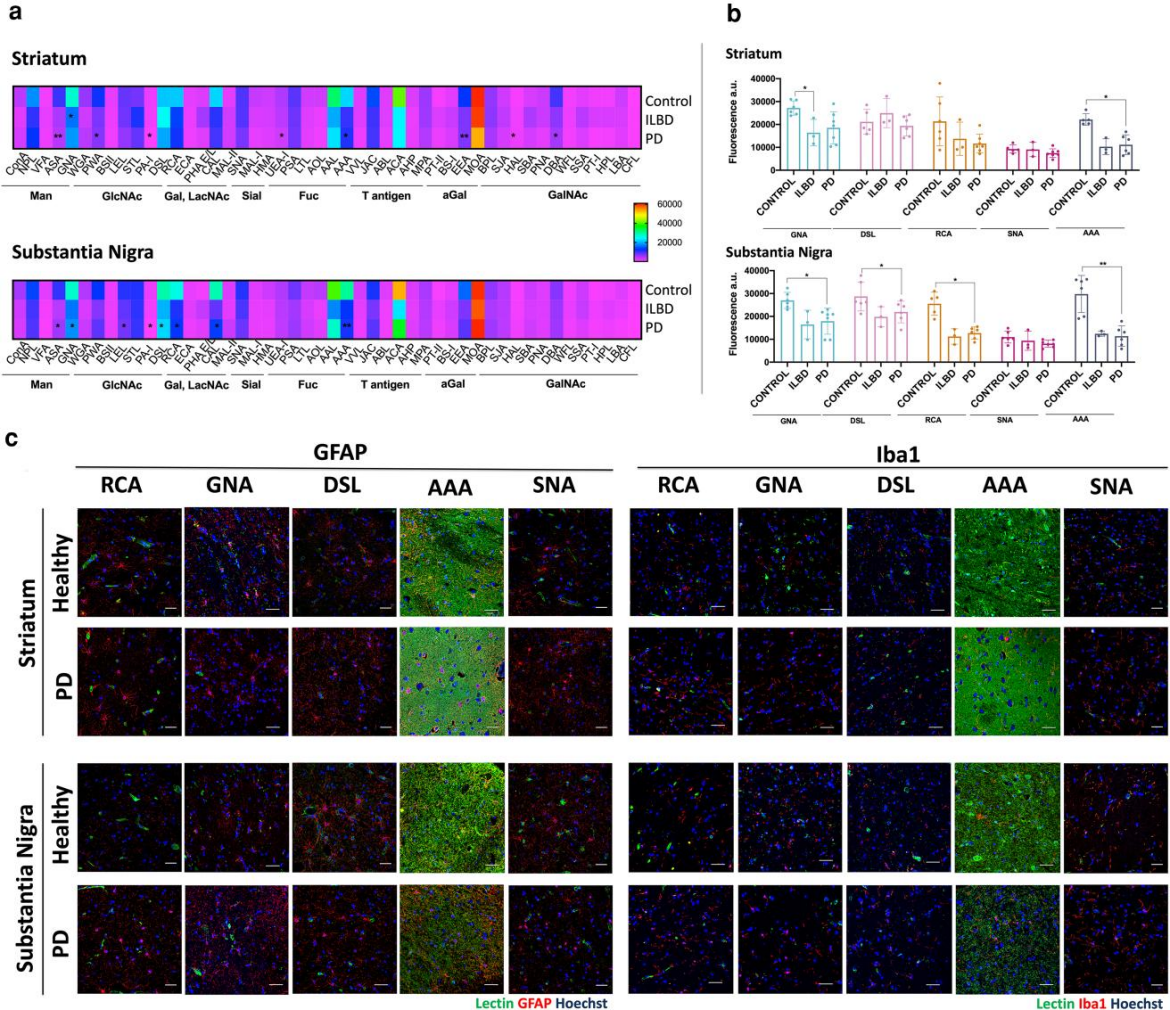
Overall changes in striatal and nigral tissue glycosylation upon PD. a) Lectin array assay performed on the protein lysate obtained from healthy (*n* = 7), ILBD patients (*n* = 3), and PD patients (*n* = 7) to detect the expression of individual glycan residues present on both *N*- and *O*-linked glycans. Two-way ANOVA was performed followed by Tukey's post hoc test and statistical significance set at *P < 0.05 and **P < 0.01 in relation to the healthy group. No significant differences were detected between the ILBD and PD groups. b) Relative expression of the main differently regulated glycans in the tissue (as indicated in the lectin array). c) Combined lectin and immunohistochemistry were used to study the spatial distribution of the main differently regulated glycans in the tissue (as indicated in the lectin array), and to associate their expression with specific inflammation-related cell types—astrocytes (GFAP+) and microglia (Iba1+). GFAP and Iba1 are labeled in red, whereas each lectin is stained in green. The nuclear stain is labeled in blue (Hoechst). Scale bar = 50 μm.

To assess the alterations happening in the overall glycome, a high-throughput screen was performed using a lectin microarray (Fig. 2). A high presence of mannose residues, multi-antennary *N*-glycans, GlcNAc oligomers, terminal galactose, α(2,6)-linked sialic acids, and fucosylated glycans were detected across groups and in both regions. Surprisingly, a low amount of Tn antigen (GalNAc-α-O-Ser/Thr) was also present, which is usually lacking in healthy cells but has been reported in the serum of Alzheimer's disease (AD) patients and their age-matched controls (21). Significant differences were detected in the binding intensity of some of these lectins, which translates into a different expression of some glycans. In the striatum, a significant decrease was seen in the binding of anguilla anguilla agglutinin (AAA) (outer arm fucose) and phytolacca americana (PWA) (branching). On the other hand, in the substantia nigra there is a significant decrease in mannosylation (allium sativum lectin [ASA] and galanthus nivalin agglutinin [GNA]), GlcNAc groups (datura stramonium [DSL], lycopersicon esculentum [LEL], and PA-I), galactosylation/poly-LacNAc groups (ricinum communis agglutinin [RCA] and cicer arietinum lectin (CAL)) as well as core fucosylation, which suggests that a decrease in complexity occurs. The binding of sambucus nigra agglutinin (SNA) and maackia amurensis lectins I and II (MAL-I/MAL-II) did not express significant differences in disease in either of the regions.

Lectin histochemistry for RCA, GNA, DSL, SNA isolectin-I, an AAA combined with immunohistochemistry for the main inflammatory-related cells (astrocytes and microglia) was performed to assess the spatial distribution of the different glycans (Fig. 2). Some of these correlated with astrocytes but not with microglia, so they seem to be mainly binding to either extracellular matrix, or other cell types (potentially neurons).

### Parkinsonian brains display region-specific alterations in *N*-glycosylation traits compared to the healthy ones

After looking at the overall glycosylation traits, a more in-depth study of the *N*-glycosylation patterns was performed, initially in healthy brains (list of the samples used is described in Table S2; healthy controls were 1:2 male to female ratio, and the ages were 79 ± 10 years). For this, a combination of hydrophilic interaction liquid chromatography–ultra performance liquid chromatography using fluorescence (HILIC–UPLC–FLR) and liquid chromatography–mass spectrometry (LC–MS) was used after assessing the reproducibility of the results with control samples (Fig. S1). The entire *N*-glycome profiles of the healthy human nigro-striatal regions are fully described in the supplementary info (Fig. S2).

The main glycosylation traits were then analyzed individually to compare the *N*-glycome between healthy, incidental Lewy bodies disease (ILBD) and PD in both regions (according to Table S1). The list of the samples used is described in Table S2: healthy controls were 1:2 male-to-female ratio, and the ages were 79 ± 10 years, PD patients were 1:1.2 male-to-female rations and ages were 80 ± 5 years. The analysis by traits rather than by individual structures is crucial since the different glyco-moieties of glycoproteins play distinct roles in facilitating the recognition, binding, and processing through the different receptors on cell membranes.

**Fig. 3.**
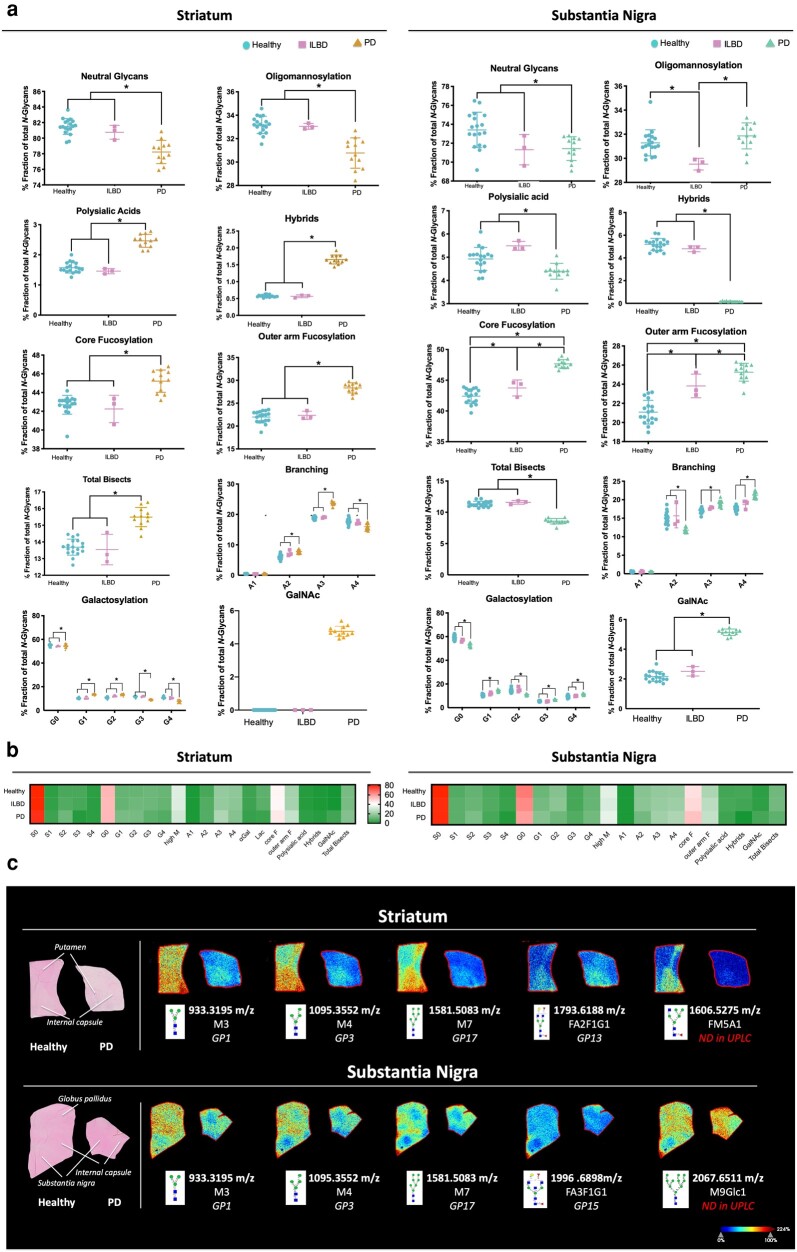
Changes in the *N*-glycosylation traits in the different regions, in health and upon disease. a) The common glycosylation features amongst the main structures in each glycan peak were grouped in the main glycosylation traits (according to Table S1). The abundance of these was log-transformed. Data is presented as mean ± SD. One-way ANOVA was performed since the data was shown to be normally distributed. This was followed by Tukey's post hoc test and statistical significance set at *P < 0.05. Blue circles: healthy; pink squares: ILBD; yellow/green triangles: PD. b) Differences seen in terms of abundance amongst the main glycosylation traits. c) *N*-glycan imaging of the main structures differently regulated in the striatum and substantia nigra of PD and age-matched control human brains. Each image is accompanied by the putative structures determined by combinations of accurate *m/z*, collision-induced dissociation (CID) fragmentation patterns, and glycan database structure. ND, nondetected.

In both regions, the abundance of neutral glycans remained similar in healthy and ILBD, decreasing significantly in the later stages of the disease (Fig. 3). Polysialic acids (PSA) were significantly increased by 1.6-fold in the striatum upon disease, however, the opposite trend was seen in the substantia nigra (11% reduction).

Concerning oligomannosylation, in the two regions analyzed the trends seen were contrasting: in the striatum the abundance of oligomannose structures seems constant between the healthy and the ILBD group, decreasing significantly at the later stages of the disease. However, in the substantia nigra there is a decrease in the presence of these glycans upon ILBD, and an increase at the later stages of the disease (Figure 3).

As for fucosylation, in both regions, a significant increase in both core and outer arm fucosylation between healthy controls and later stages of PD was seen (in the striatum, a 1.09-fold increase in core fucose and a 1.28-fold increase in outer arm fucose, whereas in the substantia nigra 1.12-fold increase in core fucose and 1.19-fold increase in outer arm fucose were seen). In the substantia nigra there is a significant increase in both types of fucosylation in the ILBD group, which suggests that fucose expression occurs in parallel with the progression of the disease. On the other hand, the expression of galactose residues in the *N*-glycan structures present both in the striatum and substantia nigra increases significantly upon PD (1.01- and 1.11-fold, respectively). Interestingly, there is a significant increase in mono-galactosylated structures in both regions (1.30- and 1.38-fold, respectively), while the tri- and tetra-galactosylated ones decrease in the striatum (21 and 32% reduction, respectively) and increase in the substantia nigra (1.28- and 1.20-fold respectively).

Regarding the branched structures, *N*-glycans with two branches are significantly increased in the striatum (1.31-fold increase), whereas they decrease in the substantia nigra. However, glycans with three branches are upregulated in both regions. Structures with four antennae are sharply decreased in the striatum and increased in the substantia nigra. Also, there are no significant changes between the healthy and ILBD groups in either the bisected or the branched glycans.

Individual *N-*glycans’ identification, validation, and spatial distribution were assessed through matrix-assisted laser desorption/ionisation (MALDI) mass spectrometry imaging (MALDI-MSI). This revealed a decreased expression of specific structures in PD brains compared to healthy ones, corroborating the significant differences already determined by HILIC–UPLC–FLR. This was the case of M3, M4, M5, and FA2F1G1 (which correspond to GP1, GP3, GP17, and GP13, respectively, on the HILIC–UPLC–FLR data) in the striatum (Fig. 3c). Another structure (FM5A1) was shown to be downregulated in PD brains through MALDI-MSI; however, this was not detected through HILIC–UPLC–FLR. This contributes to the decrease in oligomannosylation seen upon disease in the striatum. Similarly, in the case of the substantia nigra, downregulation in the expression of some oligomannose structures was also seen, mainly of M3, M4, and M7, corresponding to GP1, GP3, and GP17, respectively (Fig, 3c). A decrease in FA3F1G1 (GP15) is also seen through MALDI-MSI, and the presence of another structure (M9Glc1—increased upon PD), which was not previously detected through HILIC–UPLC–FLR. This indicates that even though there is an overall increase in oligomannosylation in the substantia nigra upon disease, the abundance of some specific structures from this glycosylation trait is decreased. Other structures were detected, however, significant changes were not identified using this technique.

The results seen highlight the possibility of using this technology not only to validate the previous data but also to assess its spatial distribution within the different regions in the striatum and substantia nigra. Furthermore, this also allows the previous data to be complemented through the detection of structures that were not deciphered previously by HILIC–UPLC–FLR and LC–MS.

### Regulation of glyco-enzymes transcripts and expression is region- and disease-dependent

As previously mentioned, glycosylation of proteins is a nontemplate-driven mechanism that depends upon the action of multiple glyco-enzymes (4). Therefore, an assessment of the changes in their transcription and expression is of significance.

**Fig. 4.**
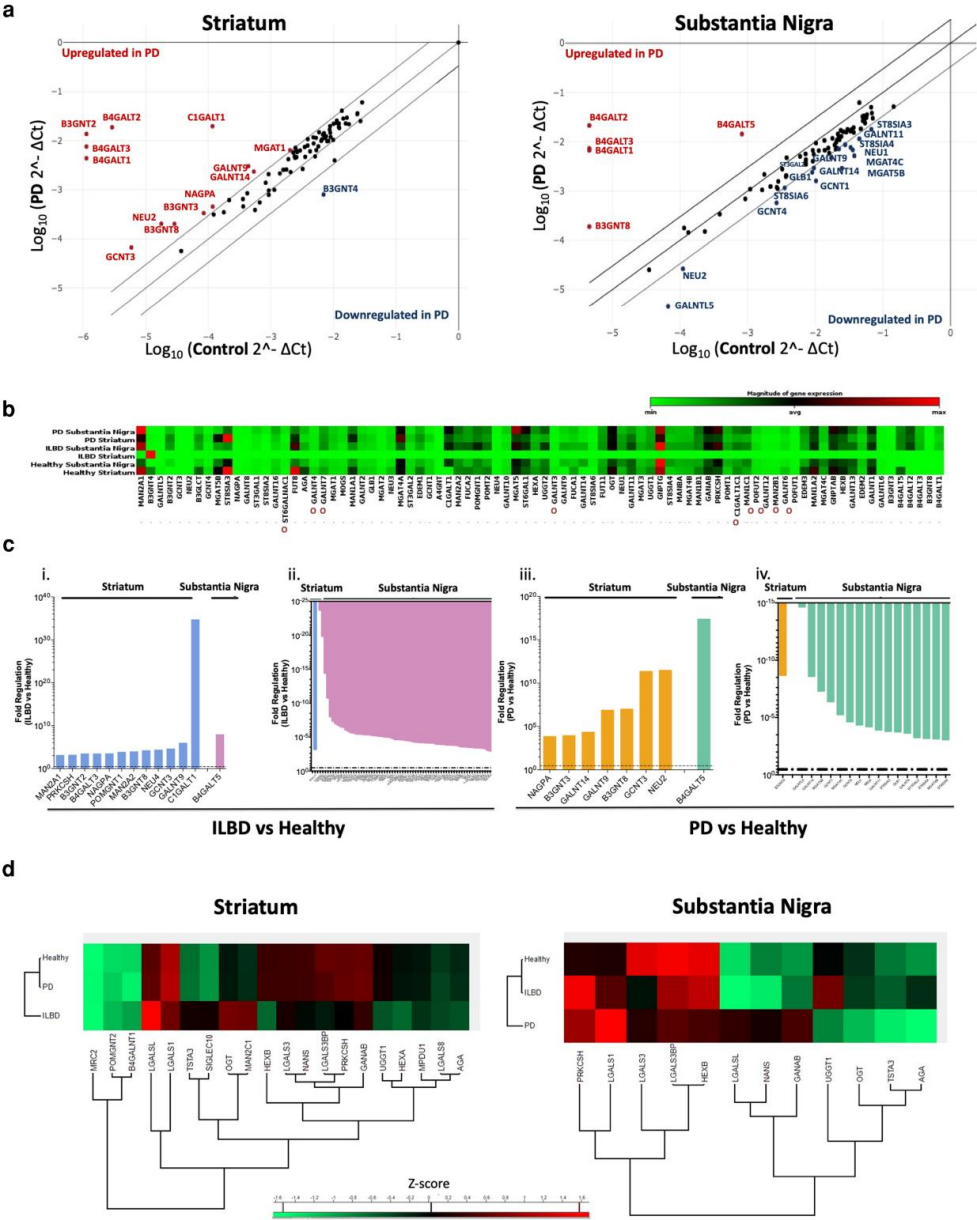
Expression of the human nigro-striatal glyco-enzymes upon ILBD and PD. a) Expression of 84 transcripts of glycosylation enzymes was performed using RT² Profiler PCR array—human glycosylation (Qiagen) and analyzed through the GeneGlobe Data Analysis Center. Significant upregulation (indicated in red) or downregulation (indicated in blue) of the different genes was considered when fold-change ≥ 3 and *P* ≤ 0.05. Data represents the pool of mRNA from six biological replicates for the healthy group, three for the ILBD group, and six for PD group. b) Relative expression of each transcript in healthy, ILBD, and PD samples. Red indicates high relative expression and green low relative expression. Expression levels were normalized on the housekeeping gene glyceraldehyde 3-phosphate dehydrogenase (GAPDH). *O* indicated enzymes involved only in *O*-glycosylation. All the others are involved only in *N*-glycosylation or in both types of glycosylation. c) Genes significantly upregulated (i and iii) or downregulated (ii and iv) in ILBD (i and ii), and in PD (iii and iv) conditions, in both the striatum and substantia nigra (considered when fold-change ≥ 3). Data represents the pool of mRNA from six biological replicates for the healthy group, three for the ILBD group, and six for the PD group. d) Relative expression of the glyco-enzymes detected by proteomics (through nanoLC–MS) in health, ILBD, and PD samples according to their *z*-score (red: upregulated proteins and green: downregulated proteins). Significant differences were considered when *z*-score ≥ 1.65 (or *P* ≤ 0.1).

In the substantia nigra of PD patients, significant downregulation was mainly seen in *N*-acetylgalactosaminyltransferases (GANTL-5, -9, -11, -14, which are predominantly involved in *O*-glycosylation, were decreased by −14.65, −3.64, −3.91, and −8.59-fold, respectively), in *N*-acetylglucosaminyltransferases (MGAT-4B, -4C, and -5B, which play a role in the branching of *N*-glycans), in sialidases (neuraminidases NEU-1 and -2) and sialyltransferases (ST8SIA-3, -4, and -6, which are mainly regulating the formation of PSA). There was also a -3.75-fold significant decrease in GLB1, a-galactosidase involved in the different types of protein glycosylation and in the processing of gangliosides, more specifically GM1, which is reported to be reduced in PD brains (22) (Fig. 4).

In the striatum, only one gene in PD was significantly downregulated—B3GNT4 (–8.69-fold reduction), an *N*-acetylglucosaminyltransferase responsible for the biosynthesis of poly-*N*-acetyllactosamine sequences. In contrast, there were multiple genes whose transcription was upregulated in this region: two *N*-acetylglucosaminyltransferases (B3GNT-3, -8, related to the elongation of branched *N*-glycans), two *N*-acetylgalactosyltransferases (GALNT-9, -14, involved in the formation of *O*-glycan core structures), and GCNT3, also involved in *O*-glycosylation (Fig. 4). The expression of a sialidase (NEU2) is also significantly increased (by 11.51-fold).

In the case of the striatum, in the ILBD group there is a significant upregulation in mannosidases expression (3.14- and 4.00-fold increase in MAN2A-1, -2, respectively, which are the final players in the *N*-glycans maturation pathway, controlling the conversion of the oligomannose structures to complex ones), and enzymes involved in *O*-glycosylation (C1GALT1, GALNT9, GCNT3, and POMGNT1) (Fig. 4). It is also important to mention the significant upregulation of N-Acetylglucosamine-1-Phosphodiester Alpha-N-Acetylglucosaminidase (NAGPA) (3.52-fold) and B3GNT-2, -8, seen both in the ILBD and PD striatum, which might indicate that the changes in these pathways start from the early stages of the disease. The expression of protein kinase C substrate 80K (PRKCSH) transcript is significantly increased also in ILBD. This is a crucial enzyme for the formation of *N*-glycans since it cleaves glucose residues from the lipid-linked oligosaccharide (LLO) formed in the *N*-glycans synthesis. Interestingly, multiple enzymes responsible for regulating the initial stages of the *N*-glycans formation are upregulated in the ILBD group. This might suggest that an adaptive response from the cellular machinery is taking place to compensate for the initial toxicity seen with the formation of the Lewy bodies. However, it is worth keeping in mind that the expression of the genes does not entirely reflect the expression of the final form of the enzymes. Also, their activity might be affected due to the pathophysiological conditions of the cellular milieu.

To assess the expression of the enzymes whose transcripts were evaluated previously, the proteomic profile was assessed by nanoLC–MS. Unfortunately, the expression of the glyco-enzymes of interest is low, so only very few of them were detected (Fig. 4d).

### Increase in specific ER stress markers is seen in the substantia nigra of PD brains

**Fig. 5.**
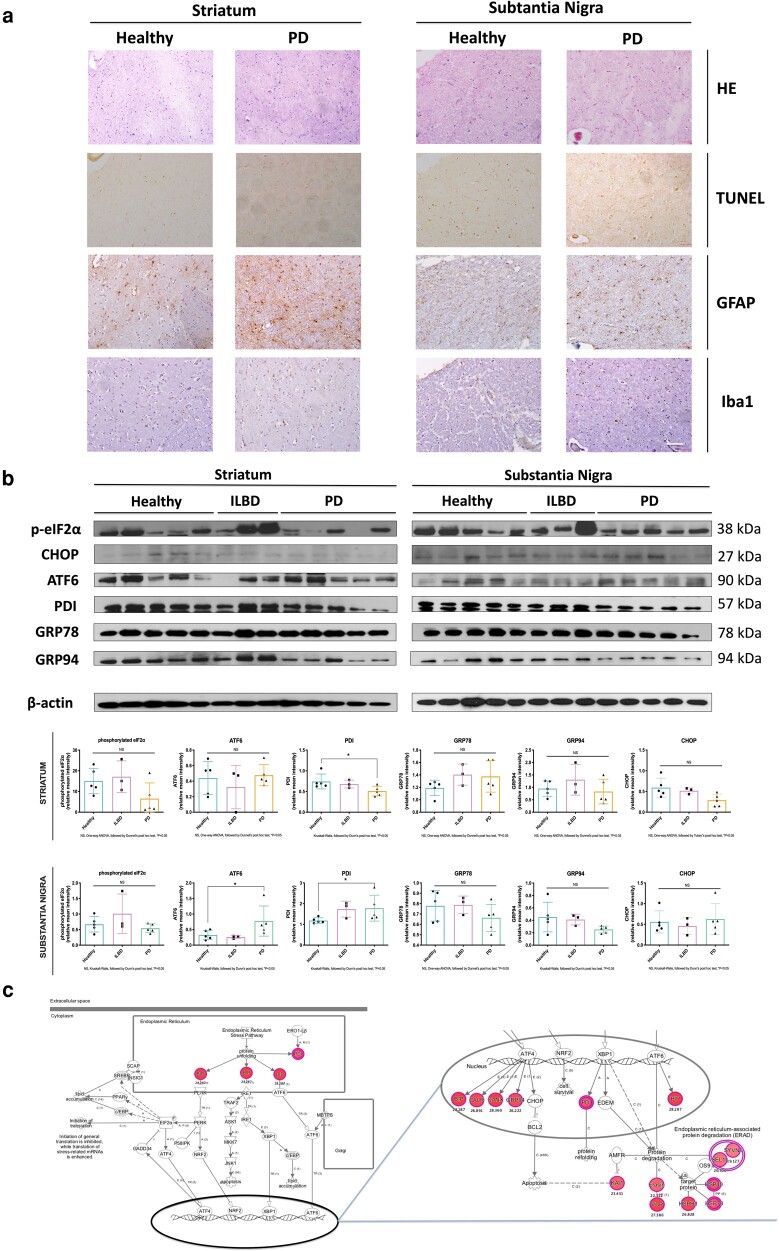
Pathological markers regulation upon PD in the striatum and substantia nigra with a focus on the ER stress and UPR. a) Morphological characterization and spatial analysis of the distribution of the main neuroinflammation-related cells in both the striatum and substantia nigra upon PD. (i) Heamatoxylin/eosin (H&E) staining, (ii) terminal deoxynucleotidyltransferase (dUTP) nick end labeling (TUNEL) staining, (iii) anti-GFAP (astrocytes marker) immunohistochemistry, and (iv) anti-Iba1 (microglia marker) immunohistochemistry. Scale bar = 100 μm. b) Expression of ER stress/UPR-related markers (GRP78, GRP94, PDI, ATF6, phosphorylated eIF2a, and C/EBP homologous protein (CHOP)) was assessed and quantified through western blot analysis. Five samples from healthy controls, three samples from ILBD patients, and five samples from PD stages 3 to 4 were considered. If the data were normally distributed, one-way ANOVA was performed and followed by the Dunnett post hoc test, and statistical significance was set at *P < 0.05. If the data were not normally distributed, Kruskall–Wallis test followed by Dunn's post hoc test was carried out, and statistical significance was set at *P < 0.05. c) Ingenuity pathway analysis (IPA) of canonical “UPR” pathway in Parkinsonian substantia nigra, based on the proteomic analysis performed after running the proteins in nanoLC–MS. Red symbols indicate identified and upregulated activation of proteins in the signaling pathway from a diseased brain associated with ER stress.

Since the ER plays a pivotal role in regulating protein homeostasis and is closely related to *N*-glycosylation, UPR was also assessed through the expression of different markers using Western blot. There were no significant changes in the markers assessed in the striatum, apart from the expression of Protein disulfide isomerase (PDI), which was significantly downregulated upon PD (31.3% reduction) (Fig. 5). In the case of the substantia nigra, there was a significant increase in the expression of partial ATF6 (2.38-fold increase) and PDI (1.51-fold increase)-classic markers of UPR activation (Fig. 5). The expression of other chaperones (GRP78 and GRP94) was not significantly altered upon disease in either of the regions studied using western blot. However, when the proteomic analysis was performed using nanoLC–MS, an upregulation was found in these chaperones and PDI in the substantia nigra, indicating an overall upregulation in this canonical pathway (Fig. 5). The undetected changes seen through western blot can be due to the defective binding between the antibody and the antigen present in each of these proteins.

### Downregulation in the expression of sialyltransferases leads to impairments in cell metabolism, changes in glyco-profile, and increase in ER stress (proof-of-concept in vitro correlation study)

The characterization of the nigro-striatal *N*-glycome, the regulation of the glyco-enzymes involved in such phenomenon, and the changes in the ER homeostasis provided in the previous paragraphs describe important information regarding the glyco-profiling of PD human brains. However, it is difficult to understand whether glycosylation is acting as a cause or an effect in the onset of neurodegenerative cascades. In fact, it is highly likely that it acts as both, promoting a cyclic chain of events. It was already reported that neuroinflammation leads to changes in glycosylation (23, 24). Nonetheless, the opposite is yet to be demonstrated. Deciphering this can be a daunting task, so we carried out a small in vitro functional study as a “proof-of-concept” highlighting how a change in glycosylation can lead to other molecular alterations in the PD context. Moreover, we emphasize how all these molecular events are connected and could potentially be targeted in future therapies.

**Fig. 6.**
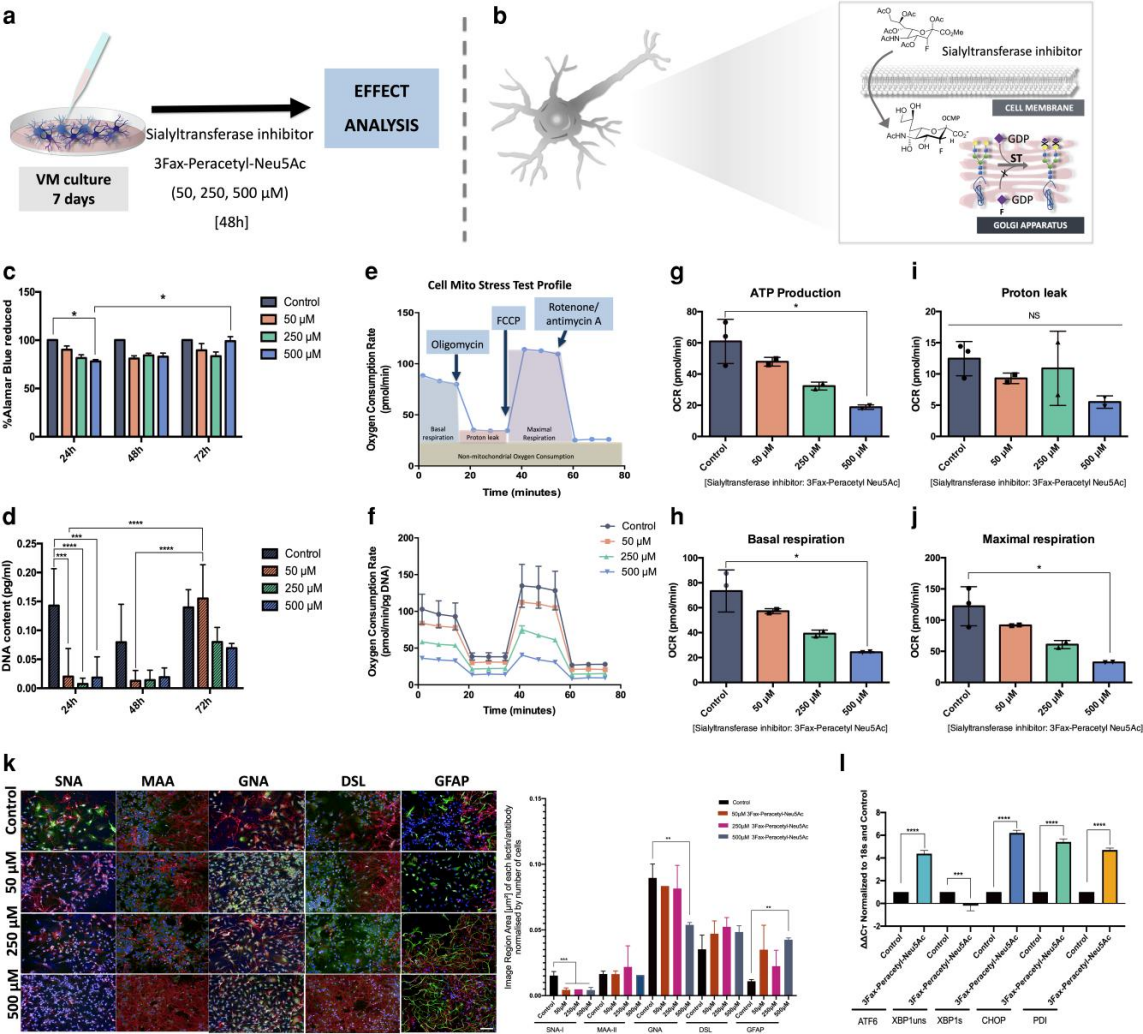
In vitro correlation study: influence of the downregulation of sialyltransferases in the cell metabolism, glyco-profile, and ER stress. a) Schematic outline of the in vitro model, time points and concentrations of 3Fax-Peracetyl Neu5Ac used, b) mechanism of action of 3Fax-Peracetyl Neu5Ac—it crosses the cell membrane and inhibits the attachment of sialic acid residues to glycan structures in the Golgi apparatus, c) cell viability of ventral mesencephalic (VM) cells when in contact with 3Fax-Peracetyl Neu5Ac assessed through alamarBlue assay (*n* = 3, data presented as the mean ± SD). Two-way ANOVA was performed, followed by Tukey post hoc test, and the statistical significance was set at *P < 0.05, **P < 0.01, ***P < 0.001, d) cell proliferation of VM cells when in contact with 3Fax-Peracetyl Neu5Ac studied through PicoGreen assay (*n* = 3, data presented as the mean ± SD). Two-way ANOVA was performed, followed by Tukey post hoc test (*P < 0.05, **P < 0.01, ***P < 0.001); e–j) Mitochondrial activity of VM cells when in contact with 3Fax-Peracetyl Neu5Ac assessed through SeaHorse Cell Mito Stress Test (Agilent, USA). In (e) the standard profile in a SeaHorse test is described, and in (f) the oxygen consumption rate (OCR) profile after each compound is added to the media is described (function of each compound is described in the materials and methods). In (g–j) the adenosine triphosphate (ATP) production, proton leak, basal respiration, and maximal respiration are described for the VM cells when in contact with different concentrations of 3Fax-Peracetyl Neu5Ac (*n* = 3, data presented as the mean ± SD). One-way ANOVA, followed by Tukey's post hoc (*P < 0.05). It shows that only the highest concentration of 3Fax-Peracetyl Neu5Ac decreases significantly the cell’s ATP production, basal respiration, and maximal respiration, k) combined lectin (in green) and immunohistochemistry (BIII-tubulin antibody in red) was used to study the expression of the main types of glycans in the VM cells upon contact with different concentrations of 3Fax-Peracetyl Neu5Ac at 48 h and to associate their expression with specific neurons (BIII-Tubulin+). Scale bar: 100 μm, one-way ANOVA was performed followed by Holm–Sidak's post hoc test (**P < 0.01 and ***P < 0.001)—A strong decrease in SNA-I binding is seen (sialylation) as well as in GNA (mannosylation), there is also an increase in GFAP (astrocytes expression), showing a upregulation in inflammation, l) mRNA expression of different UPR players is described in controls (VM cells alone with the vehicle) and cells incubated with the highest concentration of 3Fax-Peracetyl Neu5Ac (500 μM). ATF6, XBP1s, CHOP, and PDI show significant increases upon decreases in sialylation. One-way ANOVA, followed by Tukey's post hoc (*P < 0.05, **P < 0.01, ***P < 0.001).

As glycosylation is a very complex and sophisticated phenomenon, for this study, the only glycosylation trait considered was the decrease in poly sialylation in the substantia nigra, as well as the decrease in sialyltransferases (ST8SIA-3, -4, -6—mainly involving the formation of PSA) seen upon disease in this brain region. To assess whether it is the downregulation of these enzymes that could be leading to the changes in the glycome, cell homeostasis, and ER stress, a mixed primary cell culture model using rat embryonic cells from the ventral mesencephalon (origin of dopaminergic neurons) was used (VM cells), and a glycosyltransferase inhibitor (more specifically a sialyltransferase inhibitor) was employed at different concentrations (50, 250, and 500 μM). After 48 h of incubation, it was seen that the inhibition of these enzymes (sialyltransferases) led to changes in the expression of different glycans (decrease in sialylation and increase in mannosylation), in the mitochondrial activity of the cells (showing that neuronal degeneration is occurring) (in a dose-response fashion) and also in the dysregulation in the expression of UPR genes (increase in the mRNA expression of X-box binding protein 1 [XBP1] spliced, ATF6 and PDI) at the highest concentration (Fig. 6). An increase in inflammation was also seen upon the decrease in sialylation, as is shown by the increase in glial fibrillary acidic protein (GFAP) expression (reactive astrocytes). This emphasizes the impact that glycosylation can have in diseased states and how it can lead to a cascade of molecular events.

## Discussion

**Fig. 7.**
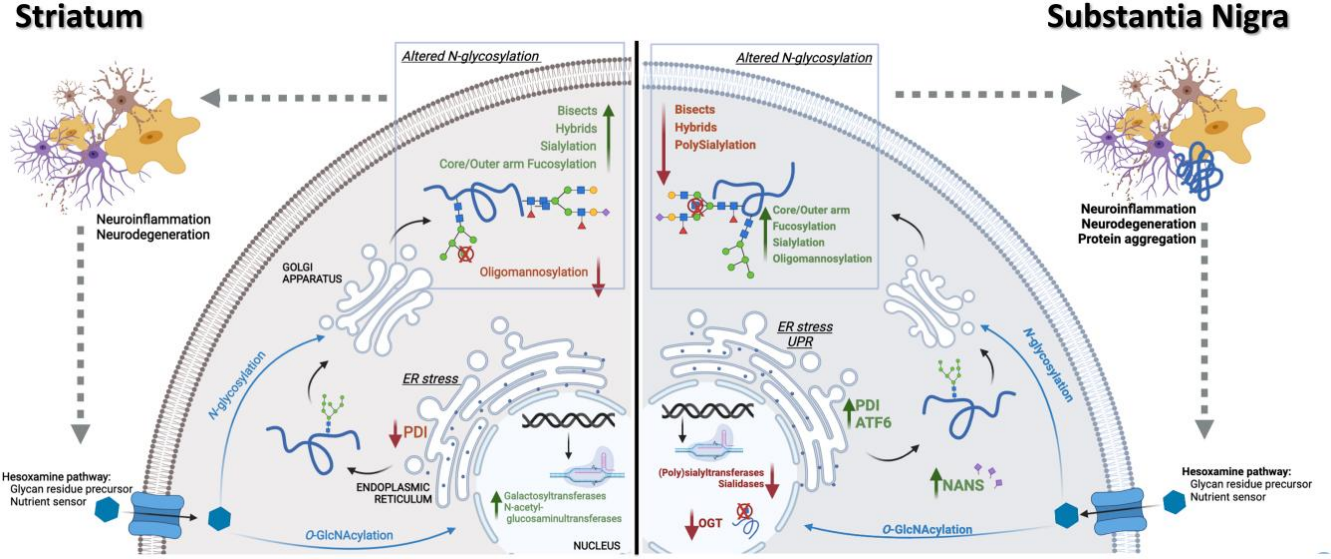
Summary and main conclusions regarding the molecular signature in each brain region upon PD. Differential regulation of glycosylation traits, glycosylation enzymes, and UPR-related proteins is seen in the striatum vs. substantia nigra. Created with BioRender (biorender.com).

The focus of this study was to characterize the protein glycosylation profile upon PD by studying the changes in the different *N*-glycosylation traits, which complements the “glyco”-studies already published on the dysregulation of GAGs (19) and the *O*-glycome (20) in human brain PD samples. *N*-glycosylation traits were analyzed in parallel and correlated with the alterations seen in the transcriptomic and proteomic expression of glyco-enzymes and the UPR in the two regions studied and in the two diseased conditions (ILBD and PD Braak stages 3/4) (overall results seen are summarized in Fig. 7).

The overall high abundance of fucosylated and oligomannosylated structures seen through the lectin array corroborates what was reported previously (25, 26). This array also showed the presence of GalNAc-containing *O*-glycans, being in accordance with the study exploring the *O*-glycome of PD brains (20). Some significant changes were seen in the substantia nigra, where a decrease in the expression of lectins binding to mannose, galactose, and GlcNAc was detected upon PD. This is a methodology that allows for high-throughput analysis, being of use as a preliminary assessment of glycomic alterations. However, it is worth noting that lectins have a broad range of ligands, so they can be used to detect carbohydrates in both glycoproteins and glycolipids, failing to show any distinction in the nature of the glycosylated molecule (27). There are also issues related to the accuracy of quantification and the possibility of unspecific binding leading to noise signal. Because the focus of this study was to elucidate specifically the *N*-glycome of the human brain, further analyses were performed using an optimized multifaceted approach combining liquid chromatography, exoglycosidase digestions, and mass spectrometry, which allowed elucidation of the in-depth composition of the nigro-striatal *N*-glyco-profile, and then investigation of the alterations in each glycosylation trait upon disease.

An overall low degree of sialylation seen (around 16 to 20%) accords with previous studies reporting the *N*-glycome from other human brain regions (25) and a study on the *N*-glyco-profile of the nigro-striatal pathway in the rat (28). The increase in sialic acid seen in the *N-*glycans from both regions upon PD occurs in parallel with the increase in sialylation in the *O*-glycans from the same regions (20). This is in contrast to studies on the *N*-glycome profiling of IgG from PD patients where sialylation is reduced (3). Increases in sialylation have been associated with brain tumors (29) to escape from immune checkpoints, which suggests that there are proteins constituents of Lewy bodies that are oversialylated, preventing their elimination. However, such a hypothesis will need to be further studied. A decrease in sialylation has been associated with neuroinflammatory models, mainly through the upregulation of neuraminidase 1 and 4 (NEU1 and NEU4), which constitute an essential factor for pathophysiological consequences upon inflammation (6, 23). Similar changes in the expression of neuraminidases are described in this study as an increase in the expression of NEU2 and NEU4 transcripts in the striatum upon disease. However, the overall sialylation is increased, raising the question as to whether this is due to the action of sialyltransferases or to impairments in the lysosomal degradation of such proteins (30). On the other hand, in the substantia nigra the expression of N-acetylneuraminate synthase (NANS) was significantly upregulated, indicating a higher abundance in sialic acid substrates upon PD. Additionally, the downregulation of NEU1 and NEU2 transcripts upon PD is involved with the overall increase of sialylation. However, such conclusions will require further analysis of these enzymes’ expression and activity since they were not detected through proteomics.

PSA is known to be involved in synaptic plasticity in brain development and was reported to be upregulated in the hippocampal regions of AD patients with neuronal loss and aggregation of amyloid plaques (31). The decrease seen in the PSA expression in the substantia nigra is parallel to decreased expression of the sialyltransferases ST8SIA3, ST8SIA4, and ST8SIA6 transcripts, suggesting the role of these sialyltransferases on the assembly of PSA.

Oligomannose structures are usually transient biosynthetic structures of *N*-linked glycans that in the case of the CNS are transported to the cell membrane to integrate the cellular glycocalyx as part of recognition molecules like neural cell adhesion molecule (NCAM) or an adhesion molecule on glia (AMOG) (7). In the adult brain, these are mainly present at the synapses, being the extent and quality of mannosylation regulated with synaptic maturation. Additionally, expression of mannose-binding lectins (MBL) has been reported in the brain, predominantly in astrocytes and microglia which are chief participants in controlling immune responses (32) through the lectin pathway of complement activation. An increase in mannose structure abundance suggests an increased binding to these receptors, which leads to immune cell activation and inflammatory response. PD is associated with a chronic state of inflammation and reports have shown that the expression of cytokines varies between striatum and substantia nigra, amongst healthy and ILBD and PD patients (32), enhancing the inflammatory pathology. This is further confirmed by the high expression of Iba1+ and changes in microglial morphology in our study. Additionally, since these structures are abundantly present at the synapsis, and synaptic disruption was recently reported in the PD substantia nigra through positron emission tomography (PET) imaging of synaptic vessel glycoprotein (33), one can hypothesize that these oligomannose residues-carrying proteins are incorrectly released to the extracellular space, that are recognized as foreign and binding to the MBL, which activates the complement system.

In the case of the striatum, it seems that the decrease in mannose expression accompanies the decrease in dopamine. It has been shown that dopaminergic agonists that act on adenylate cyclase-linked dopamine receptor sites induce a dose-dependent enhancement in the incorporation of mannose into glycoproteins in ex vivo models of rodent striatal cultures (34), so it can be hypothesized that a reversed effect is seen due to the depletion of dopamine in this brain region. Moreover, there is a significant increase in the expression of some mannosidases, which can trim the oligomannose structures and reduce their abundance in the diseased group.

Concerning fucosylation, its importance in *N*-glycans in the CNS requires further study. Fucose residues can be added to the glycan structures by the activity of one or more of the thirteen fucosyltransferases reported in the human genome, however, only nine are involved in the formation of *N*-glycan structures (35). None of these enzymes was detected through proteomic analysis, nor were their transcripts differently regulated, so it was impossible to conclude if their activity was involved with the detected changes in fucosylation. Models that fail to express core fucosylation (FUT8-knock out) show an impairment in hippocampal long-term potentiation, presenting a schizophrenia-like behavior and phenotype (36). This is accompanied by a significant increase in the abundance of microglia and astrocytes and their susceptibility to pro-inflammatory compounds, reaffirming that these phenotypes are due to defective neuronal physiology and dysfunctional glia populations (37). Considering this, a decrease in core fucosylation in the PD brain would be expected. But conversely in this study, an upregulation was seen. This is likely a contradiction in the understanding of the neurodegeneration pathway of PD that mediates pathfinding for neurons or direct neurite migration (35). However, this hypothesis will have to be further assessed.

As for galactose residues, these are particularly important in the healthy brain since sialylated *N*-glycans with multiple β1,3-linked galactoses and repeating units of PolyLac and a terminal galactose are abundant (38). Also, galactose residues that belong to *N*-acetyllactosamine moieties are recognized by galectins (e.g. galectin-1, -3, and -9), which have reportedly been expressed by activated microglia and astrocytes and are involved in neuromodulation in CNS pathophysiology, mainly by controlling inflammatory processes (39). Therefore, the increase in galactosylation seen was expected. Proteomic analysis showed a slight upregulation in galectin-1 expression in the substantia nigra but no changes were seen in the striatum.

The abundance of branched *N-*glycans is low, however, they are an essential post-translational modification for neuronal survival since the knock-down of *N*-acetylglucosaminyltransferase 1 (MGAT1; enzyme responsible for initiating the formation of hybrid glycans) leads to apoptosis and severe behavior impairments, tremors and premature death (40). The abundance of bisecting and branched *N*-glycans is intimately connected since both involve adding GlcNAc residues by MGAT to a mannose donor, and the existence of bisecting GlcNAc can limit the synthesis of branched structures. The presence of the bisecting GlcNAc (mediated by MGAT3) inhibits the action of other glycosyltransferases (mainly MGAT4 and MGAT5) (41). The expression of MGAT3 was described to increase in the AD human brain, reflecting an adaptive response to protect the brain as this increase occurs postaccumulation of β–amyloid and reduces Aβ production to protect against neurological degeneration (41). Even though the expression of MGAT3 transcript was not altered in PD brains, it is possible that the expression and/or activity of the enzyme is altered, so the increase in bisected glycans seen in the striatum can be perceived as being adaptive and protective.

The dysregulation of certain *N*-glycan structures was also reported through MALDI-MSI in AD brains (42). A region-specific modulation of *N*-glycans upon disease was also seen in this case. For example, FA2G1 (1647 *m/z*), FA2G2 (1809 *m/z*), and FA3G1 (1850 *m/z*) were all shown to be decreased upon AD in the hippocampus but increased in the cortex from the diseased brains (42). Although these facts were note-worthy, the protective, or adaptative role of disease of these structures remains to be elucidated. Interestingly, FMA5A1 (1606 *m/z*, decreased in the PD striatum) was also decreased in the hippocampus of the AD brains. On the other hand, FA2F1G1 and FA3F1G1 (1793 and 1996 *m/z*, respectively) were both increased in the cortex of AD brains, whereas they were downregulated in the striatum and substantia nigra (respectively) of PD brains. This indicates that these might be specific to each condition and potentially play different roles in the onset of distinct neurodegenerative diseases, also depending on the brain region where they are expressed.

Concerning the interplay between ER stress and changes in *N-*glycosylation, it has been reported that the increase in spliced XBP1 due to the activation of the inositol requiring kinase 1 (IRE-1) branch of the UPR leads to a decrease in bisecting glycans and sialylation, and increases oligomannose structures (43), which corresponds to the nigral PD *N*-glycomic signature. Here we saw changes in the ATF6 branch of the UPR and in other markers in the substantia nigra, which indicate that the UPR plays a role in the alterations seen in the *N*-glycomic profile since poly sialylation and bisects decreased significantly and mannosylation increased in this region. It was shown that ER stress is linked with dopaminergic neuronal death as different markers were shown to be significantly upregulated poststimuli with toxins such as 6-hydroxydopamine (6-OHDA) or 1-methyl-4-phenylpyridinium (MPP+), both in vitro (44) and in vivo (45). Also, UPR activation was correlated with the aggregation of α-synuclein in human samples (46). Nonetheless, most of these studies cover only a few of the players involved in UPR. Therefore, it is essential to look at the overall picture and assess the different cascades of the UPR response, mainly the ATF6 pathway, which hitherto has been overlooked. Only some of the markers were differently regulated through western blot (PDI and ATF6) in this study. However, this analysis was further complemented by proteomic analysis, where an upregulation of chaperones was seen (BiP and GRP94). The main function of PDI is to catalyze the formation and rearrangement of disulfide bonds in molecules (essential for proper protein folding). It has been reported that PDI has anti-inflammatory properties, inhibiting the lipopolysaccharide (LPS) produced by macrophages (47). Therefore, the increase in the expression of PDI seen in the substantia nigra is in line with the inflammatory profile seen and confirms an increase in UPR. Since there is also the accumulation of aggregated α-syn in these samples (demographic data supplied by Parkinson's UK Brain Bank), it confirms the link between UPR and protein aggregation (46).

The upregulation in the expression of the cleaved ATF6 in the substantia nigra also indicates the activation of another branch of the UPR. Cleavage of ATF6 was not detected through proteomics, so western blot was the best approach to assess this. This cleavage also leads to the upregulation of PDI, which explains the increase in both of these markers seen in the substantia nigra of PD patients (48). In a rodent model of PD (MPP+) it was shown that ATF6 was significantly increased postinjury, and that animals deficient in ATF6 displayed increased loss of dopaminergic neurons (49). It is, possible, therefore, that the changes seen in the different situations relate to the dynamic and flexible nature of the UPR and/or to the techniques used to quantify these changes. In either case, it is essential to explore the different cascades and players involved in the UPR.

The physiological glycome is extremely complex and sophisticated, making it hard to fully comprehend its function and, more specifically, the role of each glycosylation trait. Thus, most studies on the functional role of glycosylation in the brain were done using gene inactivation models, where specific glycosylation enzymes (commonly glycosyltransferases) were knocked-out in in vivo models to evaluate the influence that the lack of a particular glycan residue would have on neuronal physiology (36). However, in the past decades, the use of inhibitors for these enzymes (instead of inhibiting them genetically) has emerged as a potential pharmacological tool to not only explore the biological functions of these glycosylation traits but also to be used as possible therapeutics targeting the glycome. All the above-mentioned data characterizes changes seen in the different molecular pieces of the PD puzzle; nonetheless, it is still hard to properly correlate them. As an attempt to understand further, and since the nigro-striatal glycome is also very complex and a plethora of players were seen to be dysregulated, we opted to choose one specific glycosylation trait and test in vitro how an alteration in this trait that would correlate with the other molecular mechanisms that we have reported above. By using a sialyltransferase inhibitor (3Fax-Peracetyl Neu5Ac) and a culture of embryonic rat cells that are the progenitors of dopaminergic neurons (main cell type affected in PD substantia nigra), it was possible to clearly highlight how a decrease in the action of sialyltransferases (and subsequent sialylation and poly sialylation), also affected mannosylation (increasing it), promoted inflammation (increase in GFAP+ expression, which is a marker for astrocytic reactivity), affected the mitochondrial activity of these cells (promoting neurodegeneration) and also led to an increase in UPR markers expression. These individual alterations seen were also the ones we described previously to be seen in the human substantia nigra upon PD (where poly sialylation is decreased). Despite not having a decrease in “overall” sialylation in this region (only in poly sialylation), this small “proof-of-concept” study emphasizes that the changes seen in the expression of glycosylation enzymes could be the ones promoting the other molecular alterations. Nonetheless, it is noteworthy that this model is limited since it is only a 2D model and is only covering glycosylation changes in one single glycosylation trait, which does not even allow us to discriminate amongst the different types of sialylation. Understandably, there are many other traits involved and a clear understanding of which are the upstream vs. downstream players in the overall signature of PD is still far from being clarified. Further development of more specific inhibitors or other molecules might help to clarify some of these questions in the future.

To sum up, this study presents for the first time a comprehensive overview of the different “omic” branches of PD that relate to *N*-glycosylation in a region-dependent manner, providing information on how they interplay with disease progression, exploring pathways that were previously overlooked (Fig. 7). While it is naive to assume that these dysregulations are directly correlated, it is still a valid starting point that was not considered to date. Having this overview of how the *N*-glycome is altered in parallel with the other pieces of the PD molecular signature such as the expression of glyco-enzymes, the UPR, and the proteomic profile can help to preliminarily establish a pattern in a nontemplate-driven process. Further studies on how ER stress in the dopaminergic circuitry might be leading to *N*-glycosylation dysregulation and the investigation of single-cell glycoproteomics will be of utmost interest.

### Limitations of the study

As previously mentioned, the complexity and sophistication of the brain, as well as of the glycome, makes it challenging to study these together and to establish undeniable correlations. Therefore, there are several technological limitations associated with this study which can potentially be addressed in the future with the development of next-generation technologies. Firstly, we agree that lectin arrays and lectin staining have significant shortcomings, mainly that lectins can bind only to sugar residues (e.g. fucose, mannose, or sialic acids), so they do not discriminate between *N*- and *O-*glycans. Additionally, while they bind some motifs preferentially, they also have an affinity for other motifs and thus lack specificity. Therefore, the lectin array and lectin histochemistry were used as a preliminary fast and high-throughput assessment of the overall glycan expression in the tissue—to assess if any significant changes were seen at this point, which could potentially be used in the future as biomarkers. Due to their broadness and nonspecificity for individual glycans, we then conducted a more thorough and specific analysis of *N*-glycans using UPLC and LC–MS since this would be more accurate. Hence, we considered all methodologies important for the study: the lectin array for a preliminary assessment, lectin histochemistry for the spatial distribution of these glycans, and then the liquid chromatography/mass spectrometry and MALDI imaging for a thorough characterization.

It is also worth observing that single-cell glycomic analysis would be of paramount interest since it would allow for a specific investigation into in which type of cells these changes are occurring and would help to further elucidate the mechanisms occurring. However, this is still highly complex since the technologies available do not reach such resolution and sensitivity. For instance, the limited spatial resolution of the MALDI-MSI system used translates into the acquisition of spectra that encompass molecular information from multiple adjacent cells, preventing cellular resolution (resolution of 100 μm and large laser beam diameters [around 5 to 10 μm]). This is the same reason why even if we performed immunohistochemistry exactly on the same slides as we did MALDI-MSI, we would not be able to distinguish the measurements at the cellular level, especially in the case of brain tissue where different cell types densely populate its cytoarchitecture, being therefore very complex and intricate. Nonetheless, the recent development of new MALDI systems where an oversampling approach is coupled with laser postionization (MALDI-2) is being optimized and was tested preliminarily in brain tissue, which might be the ideal strategy for follow-up studies (50). Furthermore, laser beams with lower diameters and increased resolution are being developed, potentially allowing for a more detailed mapping of such glycans in tissue sections in the future (51).

Finally, the current study did not allow the tracking of the proteins in which the glycans are being modified nor the corresponding *N*-glycosylation sites where such alterations occur. This would require a glycoproteomic approach, which is only recently starting to be explored. This can provide important insights in the future, once such technologies are further developed (reviewed by Singh (52) and Chernykh and Kawahara (53)). Nevertheless, the type of analysis carried out in this study is a pioneer in proving the full *N*-glycome profile in the human striatum and substantia nigra, which can already help to funnel the potential future studies to be performed using glycoproteomics.

## Materials and methods

The study was designed for the region-specific and temporal characterization of the molecular signature in the Parkinsonian brain, with a focus on *N-*glycosylation. Two regions were analyzed (striatum and substantia nigra) from healthy subjects (*n* = 18), incidental Lewy-body disease (ILBD) patients (*n* = 3), and stages 3–4 PD patients (*n* = 15). Brain tissue from these patients was acquired either snap-frozen or in fixed-frozen sections (Fig. 1). Full and detailed methods can be found in the Supplementary material. These include lectin histochemistry, lectin microarray, mass spectrometry, and liquid chromatography-based methods for *N*-glycan analysis, gene array, western blot, proteomic analysis, pathological analysis, and cellular assays.

### Human brain tissue

Frozen autopsied striatum and substantia nigra from patients with PD (*n* = 12 (snap-frozen tissue), *n* = 6 [fixed-frozen, 10 μm thick sections]) or ILBD (*n* = 3 (snap-frozen tissue), *n* = 1 [fixed-frozen, 10 μm thick sections]), healthy matched controls (*n* = 15 (snap-frozen tissue), *n* = 6 [fixed-frozen, 10 μm thick sections]), and associated clinical and neuropathological data were supplied by the Parkinson's UK Brain Bank, funded by Parkinson's UK, a charity registered in England and Wales (258197) and in Scotland (SC037554). The gender, age, postmortem delay, duration of the disease, and Braak stages are described in Table S2. All experiments were done in accordance with the National University of Galway Ireland Research Ethic Committee guidelines, under the reference of 2020 March 18.

### Tissue homogenization

The snap-frozen brain tissue was homogenized in different ways depending on the assay performed. For the glycomic analysis, the snap-frozen brain tissue was homogenized in RIPA buffer (R0278, Sigma, Ireland) and cOmplete protease inhibitor cocktail (5056489001, Roche, Ireland, 1:25) through mechanical disruption using Qiagen TissueLyser LT (Qiagen, UK), at 4°C (40 Hz, 8 min). The homogenates were centrifuged at 16.000 × *g* for 20 min at 4°C and the supernatants were collected for further analysis. The protein concentration of the supernatants was calculated using the Pierce bicinchoninic acid (BCA) protein assay kit (23225, Thermo Fisher, UK). For the proteomic analysis, tissue was lysed in 100 mM Tris (pH 8.5) with 1% sodium deoxycholate, 10 mM Tris (2-carboxyethyl) phosphine (TCEP), 40 mM chloroacetamide, and cOmplete protease inhibitor cocktail. The samples were vortexed, boiled for 5 min and sonicated on ice. The homogenates were centrifuged at 20.000 × *g* for 10 min and the supernatants were collected for further analysis.

### *N*-glycan analysis

The isolated glycoproteins were dried in a vacuum centrifuge overnight (Savant SPD131DDA SpeedVac Concentrator, Thermo Fisher). These were immobilized in an acrylamide gel and reduced and alkylated. *N*-glycans were released using *N*-glycanase PNGase F (1239 U/mL, New England BioLabs, Inc. cat no. P0709L) and were fluorescently labeled with 2-aminobenzamide (2-AB) by reductive amination (28, 54, 55), and the excess of 2-AB was removed on Whatman 3MM paper (Clifton, NJ, USA) in acetonitrile washes (56).

Hydrophilic interaction liquid chromatography—ultra performance liquid chromatography (HILIC–UPLC–FLR) was performed using a UPLC Glycan BEH Amide Column, 130A, 1.7 μm particles, 2.1 × 150 mm (Waters, Milford, MA, USA) on an H Class Acquity UPLC (Waters, Milford, MA, USA) equipped with a Waters temperature control module and a Waters Acquity fluorescence detector. Solvent A was 50 mM ammonium formate, pH4.4, and solvent B was acetonitrile. The column temperature was set to 40°C and the sample temperature to 5°C. The method had a duration of 30 min and it was composed of a linear gradient of 30 to 47% of buffer A for 24 min at 0.561 mL/min flow rate, increasing to 70% at 25 min and returning to 30% at 27 min until the end of the run. Samples were injected with 70% acetonitrile. Samples were excited at 330 nm and fluorescence was measured at 420 nm. For each sample set, the system was calibrated using a dextran ladder of 2AB-labeled glucose oligomers (Waters), as described elsewhere (55). Full and detailed information regarding exoglicosidase digestions, weak anion exchange - high-performance liquid chromatography (WAX–HPLC), LC–MS, 1,2-diamino-4,5-methylenedioxybenzene (DMB), and MALDI-MSI *N*-glycan analysis can be found in the Supplementary material.

### Proteomic analysis

Proteins (200 μg) were digested with trypsin. Peptides were desalted using C18 Sep-Pak cartridges following the manufacturer's instructions and dried. The peptide concentration was determined using the Thermo Scientific Pierce quantitative fluorimetric peptide assay. Peptides were reconstituted in 100 mM triethylammonium bicarbonate (TEAB), and tandem mass tag (TMT) labeling was carried out on 30 μg of peptides from each individual sample. Further information can be found in the Supplementary material.

### Statistics

Data were processed using GraphPad Prism8 software and reported as mean ± standard deviation, if not otherwise stated. Comparisons among groups were performed by one-way or two-way ANOVA, followed by Tukey's multiple comparison post hoc test. One-way (or two-way) ANOVA was employed after confirming that the distribution of the sample mean was normal (Shapiro–Wilk normality test) and the variances of the population of the samples were equal (unpaired t test, f-test for homogeneity of variance).

### Study approval

All human samples were acquired from and associated clinical and neuropathological data were supplied by the Parkinson's UK Brain Bank, funded by Parkinson's UK, a charity registered in England and Wales (258197) and in Scotland (SC037554). All of these were postmortem samples, collected according to the ethical legislation in place in the UK. All experiments were done in accordance with the NUI Galway Research Ethic Committee guidelines, under the reference of 2020 March 18.

## Supplementary Material

pgad439_Supplementary_Data

## Data Availability

Raw data were generated at CÚRAM Research Centre for Medical Devices (Ireland), Vienna University of Technology (Austria), National Institute for Bioprocessing Research and Training (NIBRT), and University College Dublin (Ireland). Derived data supporting the findings of this study are included in the manuscript and/or supporting information.
