# Supplementary material for: Changes in tissue protein N-glycosylation and associated molecular signature occur in the human Parkinsonian brain in a region-specific manner
Source: PNAS Nexus. 2023 Dec 25;3(1):pgad439. doi: 10.1093/pnasnexus/pgad439 (PMC10766401; doi:10.1093/pnasnexus/pgad439)
Supplement: pgad439_Supplementary_Data [file pgad439_supplementary_data.zip › Rebelo et al SupInfo.pdf]

PNAS nexus

SUPPORTING INFORMATION FOR:

Changes in Tissue Protein *N*-Glycosylation and Associated Molecular Signature Occur in the Human Parkinsonian Brain in a Region-Specific Manner

Ana Lúcia Rebelo[1], Richard R. Drake[2], Martina Marchetti-Deschmann[3], Radka Saldova[1,4,5], Abhay Pandit[1]*

[1]SFI CÚRAM Research Centre for Medical Devices, National University of Ireland Galway, Ireland.
[2]Department of Cell and Molecular Pharmacology and Experimental Therapeutics, Medical University of South Carolina, Charleston, USA.
[3]Institute of Chemical Technologies and Analytics, Vienna University of Technology, Vienna, Austria.
[4]National Institute for Bioprocessing Research and Training (NIBRT), University College Dublin, Ireland.
[5]School of Medicine, College of Health and Agricultural Science, University College Dublin, Ireland.

*Professor Abhay Pandit, SFI CÚRAM Research Centre for Medical Devices, National University of Ireland Galway, Ireland.

Email: abhay.pandit@nuigalway.ie

Competing Interest Statement: All authors declare they have no competing interests.

Keywords: Parkinson's Disease, human brain, N-glycosylation, protein glycosylation, glycomics

This PDF file includes:

Supporting text
Supporting materials and methods
Figures S1 to S2.
Tables S1 to S6.
Legend for Dataset described in Tables S7 and S8.
SI References

Supporting information text:

***N*-Glycome profile of the healthy human nigro-striatal regions is mainly composed of galactosylated, highly fucosylated and oligomannosylated structures**

After looking at the overall glycosylation traits, a more in-depth study of the *N*-glycosylation patterns was performed (list of the samples used is described in Table S2; healthy controls were 1:2 male to female ratio, and the ages were 79 ± 10 years). For this, a combination of hydrophilic interaction liquid chromatography – ultra performance liquid chromatography (HILIC-UPLC-FLR) and liquid chromatography-mass spectrometry (LC-MS) was used after assessing the reproducibility of the results with control samples (Figure S1). The combination of both approaches enabled the identification and quantification of both low- and high-abundance of over 120 *N*-glycan isomers for both striatum and substantia nigra spread over 59 glycan peaks (GP) (Table S7, Table S8) (main glycan structures seen in each GP are described in Figure S2).

To analyse the distinct traits, the major glycan constituent of each peak was considered the representative feature of that peak and the main traits were calculated by adding the abundance of the peaks where the main glycan expressed shared the same structural feature (Table S1). The glycoproteins in the healthy striatum and substantia nigra were seen to carry a high percentage of galactosylated and core fucosylated structures (around 47% each). Additionally, approximately 81% of the total *N*-glycans are neutral in the striatum, whereas in the substantia nigra this value drops to around 73%. From the sialylated glycans, only about 2% and 5% contain polysialic acid in the striatum and substantia nigra, respectively (Figure 3). The abundance of bisected glycans is lower (around 14% in the striatum and 11% in the substantia nigra). Most of the glycans are also branched (approximately 68% in both regions) indicating a high complexity in the structures present in the nigrostriatal pathway (Figure 3).

Interestingly, alpha-galactose epitopes (Gal-α1-3-Gal) were detected (both by HILIC-UPLC-FLR and lectin array). However, since humans cannot synthesise this residue as they lack the enzyme α1,3-galactosyltransferase, this is most probably of non-human origin, being possibly contamination or due to dietary residues.

Supporting Materials and Methods

Dual lectin and immunohistochemistry

Frozen tissue sections (10 µm thick) were warmed at room temperature for at least 45 minutes before beginning the staining. Sections were washed with 0.05% Triton-X (TBS-T) in Tris-buffered saline supplemented with Ca2+ and Mg2+ (TBS: 20 mM Tris-HCl, 100 mM NaCl, 1 mM CaCl2, 1 mM MgCl2, pH 7.2) three times during three minutes with gentle shaking. Then these sections were blocked with 3% periodate-

treated high grade bovine serum albumin (pBSA) made in TBS for one hour at room temperature to block unspecific binding. The sections were then washed again three times with TBS for three minutes each wash. Afterwards, they were incubated with fluorescently labelled lectins (Table S4) prepared in TBS-T for one hour in the dark at room temperature. After washing the sections again with TBS, these were blocked again with 3% pBSA for one hour at room temperature and then incubated with primary antibodies (anti-glial fibrillary acid protein (GFAP) produced in rabbit (Dako, USA, Z0334, 1:400) or anti-Ionized calcium binding adaptor molecule 1 (Iba1) produced in rabbit (Wako, USA, 019-1941, 1:500)) overnight at 4oC. After 24h, sections were washed in TBS-T and TBS and incubated with the secondary antibody at room temperature for one hour (AlexaFluor 594 goat anti-rabbit IgG (H+L) (Invitrogen, Ireland, A-11012, 1:1000) in TBS. These were then washed with TBS-T and TBS and finally counterstained with Hoechst (ThermoFisher, Ireland, 33342, 1:2000 in TBS) for 15 minutes. Subsequently the sections were washed again in TBS and incubated with 0.2% sudan black B solution in 70% ethanol to quench lipofuscin auto fluorescence (granules consisting of oxidised lipids that accumulate with age in the human brain). After washing twice with TBS, sections were mounted using fluoromount aqueous mounting medium (Sigma, Ireland, F4680) and coverslips and imaged within three days after curing. Inhibition by the appropriate haptenic sugar was used as control for all lectins in the study.

Lectin microarray

Lectin solutions and antibody solutions were prepared in buffer (1.0mM D-glucose in PBS containing 0.01% of cy3-conjugated BSA). These dilutions was printed onto NHS functionalised glass slide. The slides containing the lectin arrays were incubated. The remaining NHS groups were quenched by placing the slides in a 30mM ethanolamine solution in borate buffer and then blocked with a 0.3 mg/mL BSA, 0.3mM Ca2+ solution in PBS-T 0.05%. The slides were directly dried by centrifugation without previously washing. All the lectins used in the microarray are listed in Table S3. To prevent possible interference from the sample buffer (RIPA® buffer (R0278, Sigma, Ireland) and cOmpleteTM Protease Inhibitor Cocktail (5056489001, Roche, Ireland)), during the labelling step the buffer was changed using 0.5mL 10kDa Amicon™ spin filters (Merck, Ireland). 30 µg of each sample was added to the spin filter and washed twice with PBS by centrifugation (Beckman Coulter Allegra X-22R Centrifuge). Glycoproteins were recovered at a final concentration of 0.3 µg/µL in PBS. A volume of 10 µL of PBS 10x was added to the 0.3 µg/µL solution of glycoprotein in PBS prepared previously (100µL). Afterwards, the solution was incubated with Alexa-555-NHS for 1h at room temperature. The excess dye was quenched by the addition of Tris buffer and the fluorescently labelled glycoprotein solutions were directly used in the lectin array analysis. The glycoprotein samples (at a concentration of 3 µg/mL) were added to the corresponding wells (previously printed on the slides) and incubated for 1h 30 at room temperature. The sample concentration was adjusted by adding lectin incubation buffer to the labelled glycoprotein solutions after labelling. Labelled-BSA and control dye samples were incubated under the same conditions as negative controls for lectin binding. Then the glycoprotein solutions were removed and the slide washed with PBS, dried

by centrifugation and scanned on a G265BA microarray scanner (Agilent Technologies). The images obtained were analysed with Pro Scan Array Express® software (PerkinElmer). Two-way ANOVA was performed, followed by Tukey's post-hoc test and statistical significance set at *$p<0.05$ and **$p<0.01$.

***N*-glycan analysis**

Exoglycosidase digestions of 2-AB labelled *N*-linked glycans

The 2-AB labelled *N*-glycans were digested in a volume of 10 µL for 18h at 37oC in 50 mM sodium acetate buffer, pH5.5 (except jack bean α-mannosidase (JBM), which was in 100 mM sodium acetate, 2 mM Zn2+, pH 5.0). The enzymes used were: Arthrobacter urefaciens sialidase (ABS), 0.5U/mL; Streptococcus pneumoniae sialidase (NAN1), 5U/mL; bovine testes β-galactosidase (BTG), 1U/mL; bovine kidney α - fucosidase (BKF), 1U/mL; almond meal α-fucosidase (AMF), 400U/mL; β-N-acetylglucosaminidase cloned from Streptococcus pneumonia (GUH), 400U/mL; JBM, 400U/mL; Streptococcus pneumoniae β-galactosidase (SPG), 0.4U/mL; coffee bean α-galactosidase (CBG), 25U/mL. Enzymes were purchased from Prozyme (San Leandro, CA, USA) (ABS, NAN1, BTG, SPG, CBG, BKF, JBH) or New England Biolabs (Hitchin, Herts, UK) (AMF, JBM, GUH). After incubation, enzymes were removed by filtration through 10kDa MWCO microcentrifuge filtration tubes (Pall Corporation, NY, USA). Digested N-glycans from the different samples were analysed by HILIC-UPLC-FLR (3).

Liquid chromatography-mass spectrometry (LC-MS) of *N*-linked glycans

For liquid chromatography fluorescence quadrupole time-of-flight mass spectrometry (UPLC-FLR-QTOF MS) analysis, the dried N-glycan samples were reconstituted in 3 µL of milliQ water and 9 µL acetonitrile. Online coupled fluorescence (FLR)-mass spectrometry detection was performed using a Waters Xevo G2 QTof with AcquityTM UPLC (Waters Corporation, Milford, MA, USA) and BEH Glycan column (1.0 x 150mm, 1.7 µm particle size). Sample injection volume was 10 µL. The flow rate was 0.150 mL/min and column temperature was kept at 60°C. Solvent A was 50 mM ammonium formate (pH 4.4) and solvent B was acetonitrile. A 40 minute linear gradient was used and was as follows: Solvent A at 28% for one minute, increase from 28% to 43% for 30 minutes, increase from 43% to70% for one minute, constant at 70 % for three minutes, decrease from 70% to 28% for one minute and constant at 28% for four minutes. To avoid contamination of the system, the flow was sent to waste for the first 1.2 minutes and after 32 minutes. The fluorescence detector settings were as follows: λexcitation: 320 nm, λemission: 420 nm; data rate was 1 pts/second and a PMT gain = 10. For mass spectrometry (MS) acquisition data, the instrument was operated in negative-sensitivity mode with a capillary voltage of 1.8 kV. The ion source block temperature was set at 120oC and the nitrogen desolvation gas temperature was set at 400°C. The desolvation gas had a flow rate of 600 L/h. The cone voltage was

kept at 50 V. Full-scan data for glycans were acquired over m/z range of 450 to 2500. Data was collected and processed using MassLynxTM 4.1 software (Waters Corporation, Milford, MA, USA).

Weak anion exchange – ultra performance liquid chromatography (WAX-UPLC-FLR)

WAX-UPLC-FLR was carried out using a DEAE anion exchange column, 10 μm particle size, 75 x 7.5mm on a Water AcquityTM UPLC separations module complete with a Waters AcquityTM UPLC fluorescence detector. Solvent A consisted of 20% v/v acetonitrile in water and solvent B was 0.1M ammonium acetate buffer, pH7.0, in 20% v/v acetonitrile. This method had a duration of 30 minutes and the gradient conditions were a linear gradient of solvent A from minute 5 to minute 20 from 100% to 0%. At minute 23 solvent A returns to 100%, remaining constant until the end of the run, all at a 0.750 mL/min flow rate. Samples were injected into water. Samples were excited at 330 nm and fluorescence was measured at 420 nm. For each sample set, the system was calibrated using N-glycan extracted from fetuin (3).

Glycan nomenclature

All *N*-glycans share the same pentasaccharide core composed of two core GlcNAcs linked to three mannoses. F at the start of the abbreviation indicates a core α(1,6)-fucose linked to the inner GlcNAc. Otherwise, F indicates an outer arm α(1,3) or α(1,4)-fucose linked to antenna or galactose. Mx indicates the number (x) of mannose residues on the core GlcNAcs. Ax refers to the number (x) of GlcNAc (antenna) on the trimannosyl core. Gx relates to the number (x) of β(1,4)-linked galactose on the antenna and Galx to the number (x) of α(1,3/4/6)-linked galactose on β(1,4)-linked galactose. Sx stands for the number (x) of sialic acids (neuraminic acids) linked to galactose, through an α(2,3)-, α(2,6)- or α(2,8)-linkage depending on the number inside the parentheses (3, 6 or 8, respectively). Sgx concerns the number (x) of glycolylneuraminic acids linked to galactose, and the number in parentheses corresponds to the linkage as before. Lacx indicates the number (x) of poly-N-Acetyllactosamine repeats containing GlcNAc linked β(1,4)- to galactose.

DMB assay

To assess the type of sialic acids present in the brain glycoproteins, a kit was used to release the sialic acids and label them with 1,2-diamino-4,5-methylenedioxybenzene Dihydrochloride (DMB). After homogenising the tissue as described previously, the glycoprotein solution was dried in a SpeedVac (Savant™ SPD131DDA SpeedVac™ Concentrator, ThermoFisher) overnight. On the next morning, 25 μL of 2M acetic acid solution was added to each sample, to the controls and the standards. These were briefly vortexed and centrifuged. Then they were incubated at 80oC for two hours and cooled to room temperature afterwards. 5

µL from each vial was kept at -20oC for the following steps. For the sample labelling, 440µL of the mercaptoethanol solution was added to the vial of sodium dithionite and mixed until dissolved. This solution was then added to the DMB dye and mixed. Afterwards, 20µL of this labelling reagent was added to each sample, controls and standards, mixed thoroughly and briefly centrifuged. Then the vials were incubated for three hours at 50oC in the dark. The reaction was terminated by the addition of 475 µL of water to each sample and control or of 480 µL of water to each sialic acid standard.

Ultra-high performance liquid chromatography (UHPLC)

UHPLC was performed to analyse these samples using the LudgerSepTM uR2 UHPLC column, 175 A, 1.9 µm silica derivatised particles with octadecylsiliane coating, 2.1 x 100mm (Luger, UK) on an Acquity system. Solvent A was acetonitrile:methanol:water (9:7:84) and solvent B was acetonitrile. The sample temperature was set to 10oC and the column temperature to 30oC. The method had a 15 minute duration, the flow rate was constant at 0.25 mL/min. During the first 7 minutes, the flow was solely composed of solvent A. This was reduced to 10 % (and solvent B increased to 90%) between 7.5 and 8 minutes, and returned to the initial values between minute 8.5 and 15. N-acetylneuraminic acid quantitative standard, N-glycolylneuraminic acid quantitative standard and a sialic acid reference panel containing Neu5Ac, Neu5Gc, Neu5,7Ac2, Neu5Gc,9Ac, Neu5,8Ac2, Neu5,9Ac2 and Neu5,x,xAc3 (where x is an unknown acetyl position) were used at the beginning of the run to calibrate the system. All of these were included in the kit. A negative control with only buffer (without sample) was also used.

Matrix-assisted laser desorption/ionisation mass spectrometry imaging (MALDI-MSI) of *N*-glycans

For spatial *N*-glycome analysis through MALDI mass spectrometry imaging, frozen 10 µm brain tissue sections on SuperFrostTM Plus Adhesion charged slides (Fischer Scientific, Ireland) were thawed and dehydrated in serial dilutions of ethanol (70%, 90%, 100%, 100%) for two minutes each. Then, sections were incubated at 60oC for 50 minutes, followed by delipidation in Carnoy's solution twice (30% chloroform, 10% glacial acetic acid, 60% ethanol), for three minutes, and a wash in running tap water. Finally, these were incubated with 10% formalin solution neutral buffered (Sigma, Ireland) (30 minutes), followed by two washes in tap water. The samples were then air-dried and kept in a desiccator until further analysis. The transformed slides were exposed to antigen retrieval using citraconic anhydride buffer (pH3), prepared by mixing 25 µL of citraconic anhydride (Sigma, Germany) in 50 mL of HPLC grade water. Slides were incubated in this buffer in a vegetable steamer (approximately 95oC) for 30 minutes and then washed in serial dilutions of the buffer by replacing half of the buffer with HPLC grade water (three times), eventually switching it completely with water. The slides were air-dried and scanned before applying PNGase F. After antigen retrieval, slides were coated with an aqueous solution of recombinant PNGaseF (Serva, Germany) at 0.1 µg/ µL at 45 oC, using an HTX TM-

SprayerTM (HTX Imaging, USA) as previously described (4). This was followed by an incubation of two hours at 37oC in a humidified chamber and placed in the desiccator until sprayed with matrix on the same day. α-Cyano-4-hydroxycinnamic acid (CHCA) matrix was prepared fresh (7 mg/mL in 50% acetonitrile 0.1% TFA) and applied in the sections at 100 µL/ min at 79 oC using an HTX TM-SprayerTM. Coated slides were stored in a desiccator until being analysed (no longer than 48 h post-spraying).

Gene array

Brain tissue from the striatum and substantia nigra was homogenized and lysed in TRI Reagent® (Sigma, Ireland). The RNA was extracted from this, and its concentration, purity and integrity were checked using the NanoDropTM 2000c Spectrophotometer (Thermo Scientific, UK) (reading at 260/230 nm and 260/280 nm of wave length), and the BioAnalyzerTM 2100 (Agilent, USA). cDNA synthesis was performed using the RT2 first strand kit (Qiagen, UK) and following the manufacturer's instructions.

cDNA was labelled with SyBR Green using an RT2 SyBR Green Mastermix provided by the manufacturer, which contained a HotStartDNA® Taq Polymerase (Qiagen, UK). This mixture (containing the labelled cDNA) was dispensed into the 384-wells of each plate (10 uL per well) containing 86 primers for the genes encoding for glycosylation genes such as glycosidases and glycosyltransferases (Table S5). The plate was inserted in the Roche LightCycler® 480 (Roche Life Science, Switzerland) and the cycles were defined as follows: 1 cycle for ten minutes at 95oC, and 45 cycles of 15 seconds at 95oC and one minute at 60oC. The data acquired was analysed using the GeneGlobe Data Analysis Centre® (Qiagen, UK). Significant upregulation or downregulation of the different genes was considered when fold change ≥ 3 and p ≤ 0.05. The collected data represents the pool of mRNA from six biological replicates for the healthy group, three for the ILBD group and six for the PD group.

Western blot

Protein lysates from brain samples previously homogenised in RIPA buffer (R0278, Sigma, Ireland) and cOmpleteTM Protease Inhibitor Cocktail (5056489001, Roche, Ireland, 1:25) were boiled at 95oC for seven minutes with loading buffer containing 1% bromophenol blue and 200mM of DTT. Equal amounts (5 µg or 10 µg, depending on the detection antibody) of protein samples were run on an SDS polyacrylamide gel with 12% or 15% acrylamide depending on the size of the proteins to be detected. After the run, the proteins were transferred onto a 0.45 NC nitrocellulose membrane (Fisher Scientific, Ireland) using the Trans-Blot® TurboTM transfer system (Biorad, UK). Ponceau red S staining was performed to confirm the presence of the proteins in the membrane. The ponceau red was washed off with 5% acetic acid and then the membrane was washed with TBS-T (tris buffered solution with 0.05% Tween20). The membrane was blocked with 5% milk in TBS-T for one hour at room temperature. This was followed by incubation with primary antibody for GRP94 (Cell

Signalling Technology, The Netherlands, 2104S, 1:1000), PDI (Cell Signalling Technology, The Netherlands, 3501S, 1:1000), GRP78 (Abcam, UK, ab32618, 1:3000), ATF6 (Abcam, UK, ab37149, 1:750), phospho-eIF2α (Cell Signalling Technology, The Netherlands, 9721S, 1:1000), ATF4 (Cell Signalling Technology, The Netherlands, 11815S, 1:1000) and CHOP (Cell Signalling Technology, The Netherlands, 2895S, 1:1000) overnight at 4oC. On the next day, the membrane was washed three times with TBS-T and incubated with the appropriate horseradish peroxidase-conjugated secondary antibody (Goat anti-mouse IgG, Fisher Scientific, Ireland, 31430, 1:10000, or goat anti-rabbit IgG, Fisher Scientific, Ireland, 31460, 1:10000) for one hour at room temperature. After three washes in TBS-T, signals were detected using SuperSignalTM WestPico PLUS ECL (Fisher Scientific, Ireland, 34577). Quantification of the signal intensity was performed using Image Studio Lite and normalised by the expression of β-actin (which was carried out in all the blots performed). If the data was normally distributed, one-way ANOVA was performed, followed by Dunnett post-hoc test, and statistical significance was set at $*p<0.05$. If the data was not normally distributed, a Kruskall-Wallis test followed by a Dunn's post hoc test were carried out, and statistical significance was set at $*p<0.05$.

Proteomic analysis

Proteins (200 µg) were digested with trypsin at an enzyme to substrate ratio of 1:50 (w/w) for 18 hours at 37oC, and then stopped by acidification with formic acid (FA) to a final concentration of 2% (v:v). Peptides were desalted using C18 Sep-Pak cartridges following the manufacturer's instructions, dried, and the peptide concentration was determined using the Thermo Scientific™ Pierce™ Quantitative Fluorimetric Peptide Assay. Peptides were reconstituted in 100 mM Triethylamonium bicarbonate (TEAB), and tandem mass tag (TMT) labelling was carried out on 30 µg of peptides from each individual sample. Samples were distributed in the 11-plex label set. TMT labelling reagents were each dissolved in anhydrous acetonitrile. Each sample containing 30 µg of peptide in TEAB buffer was combined with its respective 11-plex TMT reagent and incubated for one hour at room temperature. Then, 5% hydroxylamine was added, and the combined sample incubated for a further 15 minutes. This mixture was then dried for further analysis.

Nano-LC mass spectrometry

The dried sample was resuspended in 5% formic acid and desalted using a SepPak® cartridge according to the manufacturer's instructions (Waters, UK). Eluate from the SepPak® cartridge was again dried and resuspended in buffer A (20 mM ammonium hydroxide, pH 10) prior to fractionation by high pH reversed-phase chromatography using an UltiMate® 3000 liquid chromatography system (Thermo Scientific). For reversed-phase chromatography, an XBridge BEHTM C18 Column (130 Å, 3.5 µm, 2.1 mm X 150 mm, Waters, UK) was used, and peptides were eluted with an increasing gradient of buffer B (20 mM Ammonium Hydroxide in acetonitrile, pH 10) from 0% to 95% over 60 minutes. The resulting fractions were dried and resuspended

in 1% formic acid prior to analysis by nano-LC MSMS using an Orbitrap Fusion Tribrid mass spectrometer (Thermo Scientific). Here, peptides in 1% (vol/vol) formic acid were injected onto an Acclaim PepMap® C18 nano-trap column (Thermo Scientific). After washing with 0.5% (vol/vol) acetonitrile 0.1% (vol/vol) formic acid, peptides were separated on a 250 mm x 75 μm Acclaim PepMap® C18 reverse phase analytical column (Thermo Scientific) over a 150 minutes organic gradient. Solvent A was 0.1% formic acid and Solvent B was aqueous 80% acetonitrile in 0.1% formic acid. Organic gradient included 7 gradient segments (1% to 6% of solvent B over one minute, 6% to 15% of solvent B over 58 minutes, 15% to 32% of solvent B over 58 minutes, 32% to 40% of solvent B over five minutes, 40% to 90% of solvent B over one minute, and then kept at 90% of solvent B for six minutes and then reduced to 1% of solvent B over one minute) with a flow rate of 300 nL/min. Peptides were ionized by nano-electrospray ionization at 2.0kV using a stainless steel emitter with an internal diameter of 30 μm (Thermo Scientific) and a capillary temperature of 275 oC. All spectra were acquired using an Orbitrap FusionTM TribridTM mass spectrometer controlled by Xcalibur® 2.0 software (Thermo Scientific) and operated in data-dependent acquisition mode using an SPS-MS3 workflow. The raw files were analysed with MaxQuant® v. 1.6.17 and searched against the latest human fasta database (version 05.2020, 20368 sequences) downloaded from UniProt. Identified peptides and proteins were filtered to 1% FDR. TMT reagent impurity correction was performed.

Data analysis: Perseus workflow and Ingenuity pathway analysis (IPA)

MaxQuant® data was exported to Perseus® software for complete proteomics analysis, where the results were log2 transformed as well as filtered to exclude any contaminants and non-significant peptides. The final data set (including the fold ratio change of each group compared to healthy and p-values) was further analysed in the Ingenuity Pathway Analysis software (IPA®, Qiagen, UK), where canonical pathways and upstream regulator data were compared by their activation z-score and associated p-value. The main proteins and pathways considered related to unfolded protein response, glyco-related proteins/enzymes and neuroinflammatory signaling.

Pathological analysis

Immunohistochemistry (chromogenic staining)

Frozen tissue sections (10 μm thick) were warmed at room temperature for at least 45 minutes before starting the staining. Sections were incubated with 0.3% hydrogen peroxidase in 70% MeOH in PBS for 20 minutes to block endogenous peroxidase. Then the sections were washed twice with PBS and blocked with 5% normal goat serum (NGS) in PBS-T (PBS with 0.2% Triton X) for one hour at room temperature. The slides were then incubated with primary antibody solution prepared in 5% NGS in PBS-T (anti-GFAP produced in rabbit (Dako, USA, Z0334, 1:400) or anti-Iba1 produced in rabbit (Wako, USA, 019-1941, 1:500)) overnight at 4 oC. On the next day the sections were washed twice with PBS and incubated with the biotinylated secondary antibody

prepared in PBS for 30 minutes at room temperature. After washing twice in PBS, slides were incubated with streptavidin ABC HRP-complex (VectorLabs®, UK, PK-6100) for 30 minutes at room temperature. Then the slides were washed and incubated with 3,3'-Diaminobenzidine tetrahydrochloride hydrate (DAB) (2 mg/mL) (Sigma, Ireland, D9015) activated with 30% hydrogen peroxide for five minutes. Finally, the sections were counterstained with Heamatoxylin Gill no.2 (Sigma, Ireland, GHS216), dehydrated in ethanol, cleared in xylene and mounted with DPX mountant (Sigma, Ireland, 06522).

Haematoxylin/Eosin

Frozen tissue sections (10 µm thick) were warmed at room temperature for at least 45 minutes before the start of staining. Sections were hydrated in serial dilutions of ethanol (100%, 95%, 90%, 70%, 50% EtOH), followed by tap water. Then the sections were incubated with Heamatoxylin Gill no.2 (Sigma, Ireland, GHS216) followed by Eosin Y (Sigma, Ireland, HT110132). Finally, the slides were dehydrated in three solutions of 100% EtOH, cleared in xylene and mounted with DPX® mountant (Sigma, Ireland, 06522).

Terminal deoxynuclotidyl transferase dUTP nick end labelling (TUNEL) assay

TUNEL assay was performed using the Kit HRP-DAB from Abcam (Abcam, UK, ab206386) and the manufacturer's instructions were followed. Briefly, the frozen sections were warmed at room temperature for at least 45 minutes, permeabilised with Proteinase K (1:100) for ten minutes and washed with TBS. Sections were incubated with 3% hydrogen peroxidase in MeOH for five minutes to block endogenous peroxidase, washed with TBS, equilibrated with TdT equilibration buffer for 30 minutes and incubated with TdT labelling reaction mix in a humidified chamber at 22oC for 1h 30. The reaction was stopped with the stop buffer and the slides were washed with TBS. The sections were then blocked with blocking buffer for ten minutes at room temperature and incubated with the conjugate solution for 30 minutes at room temperature. Slides were washed with TBS and the stain developed using DAB for 15 minutes. Finally, the sections were counterstained with methyl green, dehydrated in two solutions of 100% EtOH, cleared in xylene and mounted with DPX® mountant (Sigma, Ireland, 06522).

In vitro cell culture model

Embryonic ventral mesencephalon rat cells extraction

Ventral mesencephalic (VM) cells were isolated from day 14 embryonic (E14) Sprague-Dawley rats. Summarily, pregnant Sprague-Dawley rats were euthanised by decapitation under isofluorane, the yolk sac was removed by laparotomy, the embryos excised from the sac and the brains separated from their heads on ice-cold Hank's Balanced Salt Solution (HBSS) (Sigma, Ireland). The mesencephalon was removed from each brain, and the VM tissue was cut from this. The tissue was trypsinized, manually dissociated and

suspended in media, and the isolated cells were incubated in a humidified atmosphere of 5% CO2 at 37 oC and cultured in media (Dulbecco's modified Eagle's medium/F12, 1% penicillin-streptomycin (PS), 1% fetal bovine serum (FBS), 33 x 10^{-3} M D-glucose, 1%L-glutamine, supplemented with 2% B27). All the reagents mentioned are from Sigma, Ireland, except B27, which is from ThermoFisher, Ireland. Each experiment was done in triplicate.

Sialylation-deficient model

To assess the effect of sialylation on neuronal/glial physiology, a fluorinated sialyltransferase inhibitor was put in contact with VM cell cultures. This inhibitor competes actively with sialic acids (Neu5Ac/Neu5Gc), preventing the action of sialyltransferases in including sialic acids in glycan structures during their processing in the Golgi. After seven days in culture, 3Fax-Peracetyl-Neu5Ac (566224, Merck, Ireland) was incubated with the cells at 50, 250 or 500 µM for 48 hours in a humidified atmosphere of 5% CO2 at 37 oC. Subsequently, the cells were collected and the effects of the inhibitor on cell physiology were analysed. Further methods are described in the supplementary materials and methods.

Biological assays *in vitro*

To evaluate the effects of the sialyltransferase inhibitor on the physiology of the cellular culture, multiple assays were carried out, as described below.

Cell metabolic activity: AlamarBlue®

To investigate the cells metabolic activity at different concentrations of sialyltransferase inhibitor, alamarBlue® assay (Invitrogen, Ireland) was used. Briefly, at the pre-defined time-points, the cells were incubated with a 10% solution of alamarBlue in HBSS for three hours. Subsequently, the absorbance from the alamarBlue solution was read at 450 nm and 550 nm using a Varioskan Flash plate reader (ThermoFisher, UK). HBSS and alamarBlue (100%) alone were used as negative and positive controls, respectively, for the succeeding analysis.

DNA quantification: Quant-iT PicoGreenTM dsDNA assay

Quant-iTTM PicoGreenTM dsDNA assay kit (Invitrogen, Ireland) was used to assess the DNA content (direct measure of cell proliferation). To collect the samples, the media was removed from the cells, these were washed with HBSS, and then DNase free water was added to each well. These underwent three cycles of

freezing-thawing (alternating between at least 15 minutes at -80 °C and at least 1 hour at room temperature). Afterwards, samples were incubated with TE buffer (at 1X) and PicoGreen reagent (at 1X) for ten minutes in a 96-well plate (protected from light) and the fluorescence was read at 520 nm using a Varioskan Flash plate reader (ThermoFisher, UK).

Mitochondrial activity: MitoStress test (Seahorse extracellular flux analysis system®)

To study the cultures' mitochondrial activity in real time, more specifically the oxygen consumption rate (OCR) and associated cellular basal and maximal respiration, ATP production and proton (H+) leak, the Seahorse extracellular flux analysis system and corresponding MitoStress test (Agilent Technologies, USA) were used as per manufacturer's instructions (and following the method already published (57)). Briefly, cells were seeded at 2.5 x 104 cells/well into 8-well Seahorse XFp cell culture miniplates (Agilent Technologies, USA) and treated with the sialyltransferase inhibitor for 48 hours. Then, the culture medium was replaced with Seahorse assay medium (Seahorse Base XF medium, with 10 mM D-glucose, 2 mM L-glutamine, 1mM Na+ pyruvate, at pH 7.4) and the cells were left for one hour prior to the assay in an incubator without CO2 at 37 ºC. Mitochondrial function was investigated through bioenergetics profiles that were generated by sequential injection of multiple mitochondrial toxins resuspended in Seahorse assay medium: 1) oligomycin A (an ATP synthesase inhibitor that blocks its proton channel/complex V), 2) carbonyl cyanide-4-(trifluoromethoxy)phenylhydrazone (FCCP; an uncoupling agent that disrupts the mitochondrial membrane potential by transporting hydrogen protons through the membrane and leading the respiration rate to its maximum capacity), and 3) rotenone/antimycin A (complex I and III inhibitors, respectively, which shut down mitochondrial respiration). These were loaded in the cartridge in a way to reach the final optimised concentrations of 1.5 μM, 3 μM and 0.5 μM, respectively, in the wells. At the end of the assay, a graph and data sheet were generated in the system, allowing for the analysis of the effect of the different compounds on the mitochondrial function.

RNA isolation and gene expression

Cells were washed thoroughly with PBS before adding TRIzol®. A pipette tip was used to scrape them and then the TRIzol® solution was transferred to a 2 mL microcentrifuge Eppendorf tube. Samples were centrifuged at 1,200 ×g for 5 min at 4 °C and the cellular debris was discarded. The clear supernatant was transferred to a clean tube and 200 μL chloroform was added per 1 mL TRIzol® solution, following the protocol described before for RNA extraction from tissue. cDNA synthesis was also carried out as above-mentioned. cDNA products were amplified using Fast SYBR® Green Master Mix (Applied Biosystems®, 4385612) and following specific primers (Sigma-Aldrich) (Table S6).Reactions were conducted in triplicate

using StepOnePlus™ Real-Time PCR System (Applied Biosystems® . The results were analysed using the 2-ΔΔCt method and presented as fold change (relative gene expression) normalized to 18S and basal control.

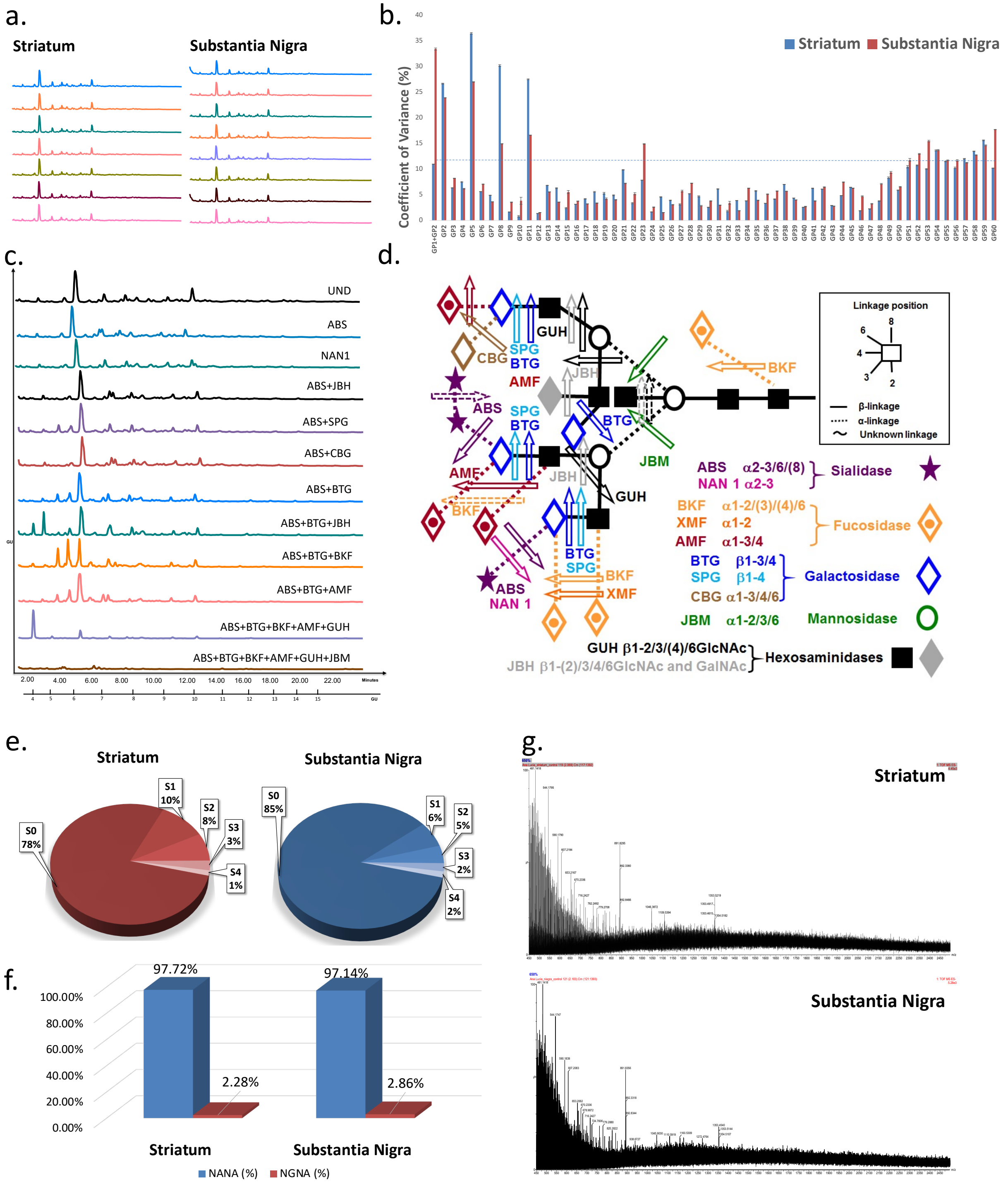

Figure S1. Development and optimisation of the glyco-analytical platform based on liquid-chromatography methods to characterise the human nigro-striatal *N*-glycome. a. Reproducibility of the platform is represented by the overlay of biological replicates, which are indicated by different colours for both healthy striatum and substantia nigra. **b.** Comparison of the individual glycan peaks (GP) area

coefficients of variance for eight samples from both striatum and substantia nigra using HILIC-UPLC-FLR. Separation of chromatograms into GP can be seen in Figure **S2**. To characterise the chromatograms obtained in each region, a sequence of exoglycosidase digestions was performed **c.** Representative chromatograms for the exoglycosidase digestions performed on the striatum samples using HILIC-UPLC-FLR (similar profiles were obtained for substantia nigra – data not shown). Through the shift of each peak after incubation with the different enzymatic cocktails, it was possible to build the glycan structures present in each of the peaks, as well as to calculate their abundance (Figure **S2**). Detailed information on all exoglycosidase digestions can be found in **Supplementary table S7**. **d.** Schematic representation of the specificity of each exoglycosidase. **e.** Percentage fractions of sialylated *N*-glycans in both striatum and substantia nigra, classified according to the degree of sialylation (mono- (S1), di-(S2), tri- (3), tetra- (S4)) using WAX-UPLC-FLR. **f.** Percentage fraction of the different types of sialylation (Neu5Ac (NANA) vs Neu5Gc (NGNA)) present in the overall pool of glycoproteins in the healthy striatum and substantia nigra using DMB assay. Human cells no longer have the ability to synthesize Neu5Gc, however it is possible that these were included in the glycan structures due to dietary incorporation (1). **g**. LC-MS profiles of the *N*-glycans from both healthy striatum and substantia nigra. All the results gathered from the exoglycosidase digestions, WAX-UPLC-FLR profile, DMB profile and LC-MS were used to characterise and describe the *N*-glycome HILIC-UPLC-FLR -based profile for each brain region, in healthy conditions and upon disease. Abbreviations: DMB: 1,2-diamino-4,5-methylenedioxybenzene Dihydrochloride, LC-MS: Liquid chromatography-mass spectrometry, HILIC-UPLC-FLR: Hydrophilic Interaction Liquid Chromatography - Ultra Performance Liquid Chromatography, WAX-UPLC-FLR: Weak Anion Exchange - Ultra Performance Liquid Chromatography.

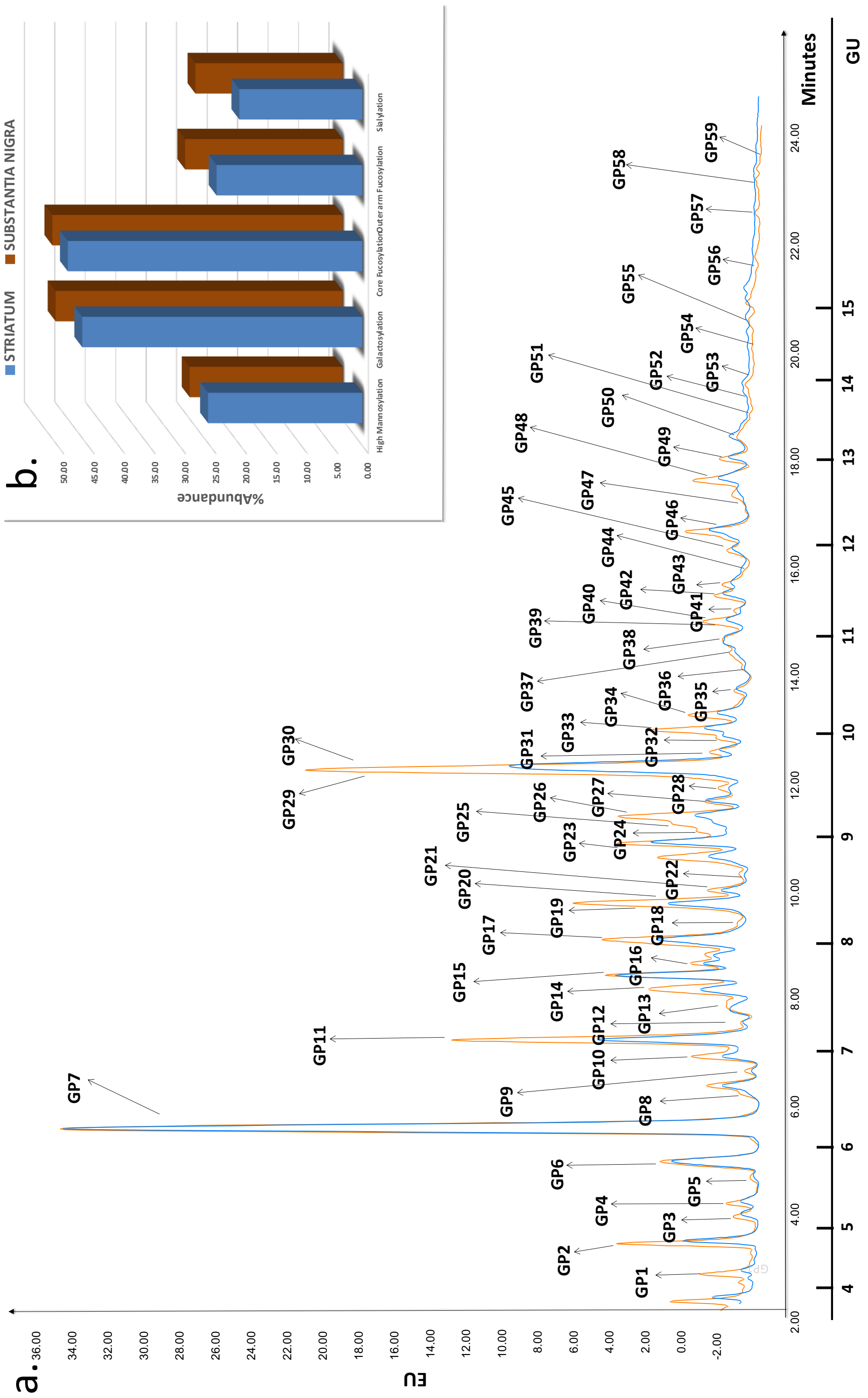
a.
EU
Minutes
GU
b.
STRIATUM
SUBSTANTIA NIGRA
%Abundance
High Mannosylation
Galactosylation
Core Fucosylation
Outer arm Fucosylation
Sialylation

c.

Peak number	STRIATUM				Peak number	SUBSTANTIA NIGRA			
	HEALTHY		PD			HEALTHY		PD	
GP	Structure	Peak Area (%)	Structure	Peak Area (%)	GP	Structure	Peak Area (%)	Structure	Peak Area (%)
1	M3	0.44	M3	0.37	1	M3	0.49	M3	0.43
2	F(6)M3	2.11	F(6)M3	1.53	2	F(6)M3	1.70	F(6)M3	1.76
3	M4	0.43	M4	0.46	3	M4	0.42	M4	0.44
4	F(6)A1	0.48	F(6)A1	0.53	4	F(6)A1	0.51	F(6)A1	0.59
5	A2B	0.78	A2B	0.20	5	M4A1	0.32	M4A1	0.31
6	A3	2.57	A3	2.53	6	F(6)A2	2.29	A3/F(6)A2	2.27
7	F(6)A3 (M5)	20.46	F(6)A3 (M5)	21.09	7	F(6)A3 (M5)	19.01	F(6)A3 (M5)	18.74
8	F(6)M5	1.21	F(6)M4A1B	1.19	8	F(6)M4A1B	1.16	F(6)M5	1.24
9	M5A1	0.15	A4B	0.16	9	A4B	0.15	A2G1GalNAc1S(3)1 (SO4- on GalNAc)	0.17
10	F(6)A4	1.05	F(6)A4	1.16	10	F(6)A4	1.08	F(6)A4	1.28
11	M6	5.09	M6	5.13	11	M6	5.81	M6	6.18
12	M5A2	0.40	M5A2	0.43	12	M5A2	0.35	A2F1G1GalNAc1	0.33
13	A3G(4)2/A3G(3)2	0.81	F(6)A2F1G(4)1	0.82	13	F(6)A2BG1	0.68	F(6)A4GalNAc1	0.62
14	M7/A4F1	1.79	M7	1.84	14	A3BG(4)2	2.02	F(6)A2G(3)2/F(6)A2G(4)2	2.07
15	F(6)A2BF1G1	3.89	F(6)A3F1G1/F(6)A2BF1G1	3.98	15	F(6)M5A2	3.37	F(6)A3F1G1/F(6)A2BF1G1	3.09
16	M7	1.46	F(6)A3F1G1	1.55	16	M7	1.36	M7/F(6)A2F1G1GalNAc1	1.35
17	M7	4.42	M7	4.42	17	M7	4.68	M7	4.72
18	F(6)A4BF1	0.95	F(6)A4GalNAc2	1.03	18	F(6)A2BG1S(3)1	0.92	M7	0.91
19	A3F3	1.18	A3F3	1.41	19	F(6)A4F1GalNAc1	1.29	F(6)A4F1GalNAc1	1.56
20	A3G(4)3	1.09	A2G2S(3)1	0.90	20	A2G2S(3)1	1.27	A2G2S(3)1	1.09
21	F(6)A4G1Lac1	1.12	F(6)A3BF1G(4)1	1.12	21	F(6)M7	1.02	A3F1G1Sg(6)1	0.98
22	A3BG(3)3/A3BG(4)3	0.50	A3BG(3)3/A3BG(3)3	0.52	22	A3BG(3)3	0.48	F(6)A2G(4)2S(3)1	0.51
23	M8	4.15	M8	4.30	23	M8	4.50	M8	4.35
24	M8	0.46	M8	0.51	24	M8	0.44	F(6)A4F2G(3)4	0.35
25	F(6)A3BG(4)3	0.95	F(6)A4F1G2	0.98	25	F(6)A3BG(4)3/A4F2G1	1.00	M8	1.02
26	F(6)A2BF2G2	1.85	F(6)A2BF2G2	1.90	26	F(6)A3F2G1S(3)1/F(6)A3F2G1S(6)1	2.02	F(6)A3F2G1S(3)1	1.89
27	A3BF2G1S(3)2/A3BF2G1S(6)2	1.86	F(6)A4F3	1.95	27	F(6)A4F3	1.72	A3BF2G1S(6)2	1.44
28	A4G(3)3Lac1/A4G(4)3Lac1	0.93	F(6)A2F2G2GalNAc1	0.89	28	F(6)A3G2Gal2	0.88	A4G(3)3Lac1/A4G(4)3Lac1	0.81
29	M9	5.05	M9	5.43	29	M9	5.31	M9	4.84
30	A2G2S(6)2	3.17	A2G2S(3)2	3.13	30	A2G2S(6)2	3.66	F(6)A2G(4)2S(3,6)2	3.87
31	F(6)A4G3Lac1	0.98	A3F1G1Gal2GalNAc1	1.05	31	A4G(4)4/A4G(3)4	1.06	A4G(4)4	1.21
32	F(6)A3G3S(3)1	0.64	N/A	0.61	32	FA4F1GalNAc1S(6,8)2	0.66	F(6)A3G3S(3)1	0.55
33	F(6)A2F2G2S(6)1	1.77	A4BG3S(3)1	1.81	33	A4F2G2	1.71	A4BG(3)4	1.57
34	F(6)A4F3G1	1.08	F6)A3G2Lac2	0.94	34	F(6)A4F3G(4)1	0.99	F(6)A4F3G1	0.96
35	F(6)A3BG2Lac2	0.78	F(6)A4G(4)4	0.85	35	A3BG3S(6)2	0.81	F(6)A4G2Sg(6)1	0.74
36	F(6)A4F1G(3)3	0.43	F(6)A4F1G(3)3	0.44	36	F(6)A4F1G3	0.40	F(6)A4F1G3	0.29
37	A4G(3)4Gal1	0.52	A4G3GlcNAc3	0.54	37	A4G4Gal1	0.51	A4G(4)4Gal1	0.55
38	M10	2.20	F(6)A3G3S(3)2	2.28	38	F(6)A3G3S(6)2	1.97	F(6)A3F2G(3)3/F(6)A3F2G(4)3	1.78
39	F(6)A3BG3GlcNAc2S(3,6)2/ F(6)A3BG3GlcNAc2S(6)2	0.78	F(6)A4F3GalNAc3	0.65	39	A4BF3G1S(6)1	1.05	A4BF3G1S(6)1	1.07
40	F(6)A4BF4G1	0.71	F(6)A4BF4G1	0.79	40	F(6)A4BF4G1	0.81	F(6)A4BF4G1	0.93
41	F(6)A4G4Gal1	0.47	F(6)A4G(4)4Gal1	0.45	41	F(6)A4G(4)4Gal1/F(6)A4G(3)4Gal1	0.57	F(6)A4F3GalNAc4	0.63
42	M11	0.97	A3F1G1Gal2S(3)2/A3F1G1Gal2S(6)2	1.02	42	M11	1.04	F(6)A4G2Sg(3)2	1.01
43	A3F1G1Gal2S(3)3	1.00	F(6)A4F1G2S(6)2	1.00	43	A3F1G1Gal2S(3)3	1.00	A3F1G1Gal2S(3)3	1.11
44	F(6)A3F1G2Sg(3,3,8)3	0.35	F(6)A3F1G2Sg(3,3,8)3	0.41	44	A4F3G4	0.30	A4F3G(4)4	0.22
45	F(6)A4G(3)4Lac1Gal1/ F(6)A4G(4)4Lac1Gal1	1.38	F(6)A4F3G1Sg(3)1	1.35	45	F(6)A4G(3)4Lac1Gal1/ F(6)A4G(4)4Lac1Gal1	1.42	F(6)A4G3Lac1Gal1	1.66
46	F(6)A4F3G3	1.60	A4BG3S(6)1Sg(6)1	1.51	46	F(6)A3BF1G2S(3,6,8)3,F(6)A3BF1G2S(3,3,8)3	2.10	F(6)A3BF1G2S(3,3,8)3/F(6)A3BF1G2S(6,6,8)3	1.49
47	A4BF3G3	0.36	A4BG3Lac1GlcNAc2S(3)1	0.32	47	F(6)A3F2G1S(3,8,8,8)4	0.80	F(6)A4F3G1S(6,8)2	1.22
48	F(6)A4F3G4	2.20	F(6)A4F(2)3G(4)4	2.17	48	F(6)A2G2Gal2Sg(6)2	1.94	F(6)A2G2Gal2Sg(6)2/F(6)A4F1G3S(3)3/ F(6)A4F1G3S(6)3	2.26
49	A3F1G(4)3S(6)2Sg(3)1	1.18	F(6)A3BG3Sg(6)2	1.08	49	A3F(3)1G(4)3S(6)2Sg(3)1	1.81	A3F(3)1G(4)3S(6)2Sg(3)1	1.54
50	A4G4S(3)4	0.40	A4G4S(3)4	0.39	50	F(6)A4BF4G1Sg(6)1/A4BF3G1S(3,8,8)3	1.99	A4G4S(3)4/F(6)A4BF4G1Sg(3)1	0.57
51	F(6)A4G(3)4Lac3/F(6)A4G(4)4Lac3	2.15	A4BF3G3GalNAc2	2.13	51	A4F4G(4)4S(3)1	0.41	A4F4G(4)4S(3)1	2.43
52	F(6)A4G4S(3)4	0.81	F(6)A4G4S(6)4	0.83	52	F(6)A4G4S(3)4 /F(6)A4G4S(6)4	0.78	A4G(3)4Gal1GalNAc2S(3)2/A4G(3)4Gal1GalNAc2S(6)2	0.65
53	A4BG4S(3)3/A4BG4S(6)3	0.82	A4BG4S(6)3	0.83	53	A4BG4S(3)3/A4BG4S(6)3	0.87	A4BG4S(3)3/A4BG4S(6)3	0.96
54	A4F3G4S(3)4/A4F3G4S(6)4	1.35	A4F3G4S(3)4/A4F3G4S(3,6,6,6)4/ A4F3G4S(6)4	1.33	54	A4F3G4S(6)4	1.19	A4F3G4S(6)4	1.04
55	F(6)A4BF4G2Gal3	0.29	F(6)A4BF4G2Gal3	0.25	55	F(6)A4G4Lac4Gal1	0.25	F(6)A4G4Lac4Gal1	0.43
56	F(6)A4G4Lac3S(6)2	1.58	F(6)A4G4Lac3S(3)2	1.55	56	F(6)A4F3G4S(3,3,6)3/F(6)A4F3G4S(6)3	1.45	F(6)A4G4Lac3S(3)2	1.33
57	A2G2S(6,6,8,8)4	0.40	A2G2S(6,6,8,8)4	0.41	57	A2G2S(6,6,8,8)4	0.40	A2G2S(6,6,8,8)4	0.40
58	F(6)A4BF2G3S(6)3	0.45	F(6)A4F3GalNAc1S(6,8,8,8)4	0.37	58	F(6)A4BF2G(4)3S(6)3	0.42	F(6)A4F2G4S(6)1Sg(3)4	1.11
59	F(6)A4F2G4Sg(3)4S(8)1	0.57	A4BF3G4S(6)4	0.54	59	A4G4Gal1GalNAc2S(6)4	0.69	A4G4Gal1GalNAc2S(6)4	0.06

d.

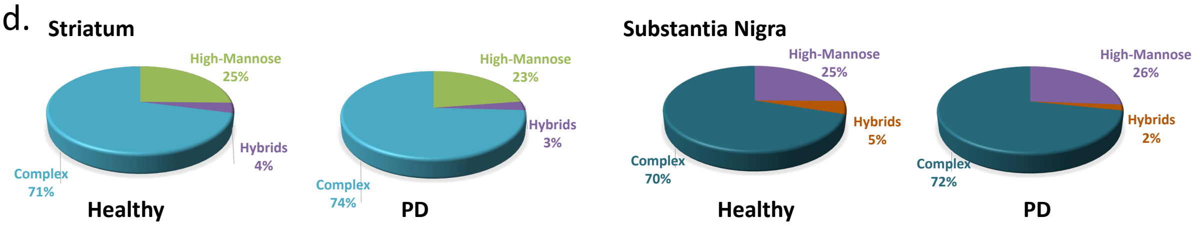

Figure S2. *N*-glycome in nigro-striatal regions of the healthy controls and PD human patients. a. HILIC-UPLC-FLR chromatograms obtained for both human striatum (blue profile) and substantia nigra (orange profile). The *N*-glycome for both these regions was separated into 59 chromatographic peaks (GP). EU – emission units. **b.** Abundance of the main glycosylation traits present in the healthy striatum and substantia nigra. **c.** Summary of the detailed composition analysis for each of these peaks, indicating the major glycan structure present in each peak and the peak area. d. Abundance of the three general types of *N*-glycans (hybrid, complex and high mannose) in both the striatum and substantia nigra, in healthy and PD samples

Table S1. Calculation of derived glycosylation traits in the striatum. The description of each glycosylation trait and the calculation rationale are presented as follows.

			Healthy/ILBD		PD	
			Striatum	Substantia Nigra	Striatum	Substantia Nigra
Charge analysis	N1	The percentage of total neutral glycans in total glycans	SUM(GP1:17,GP19:26,GP28:29,GP31,GP34:38,GP40:42,GP45:48,GP51,GP55)	SUM(GP1:17,GP19,GP21:25,GP27:29,GP31,GP33:34,GP36:37,GP40:42,GP44:45,GP55)	SUM(GP1:19,GP21:29,GP31,GP34:37,GP39:41,GP48,GP51,GP55)	SUM(GP1:8,GP10:19,GP23:25,GP28:29,GP31,GP33:34,GP36:38,GP40:41,GP44:45,GP55)
	S^{Total}	The percentage of total sialylated glycans in total glycans	SUM(GP18,GP27,GP30,GP32:33,GP39,GP43:44,GP49:50,GP52:54,GP56:59)	SUM(GP18,GP20,GP26,GP30,GP32,GP35,GP38,:39,GP43,GP46:54,GP56:59)	SUM(GP20,GP30,GP33,GP38,GP42:47,GP49:50,GP52:54,GP56:59)	SUM(GP9,GP20:22,GP26:27,GP30,GP32,GP35,GP39,GP42:43,GP46:54,GP56:59)
	S1	The percentage of mono-sialylated glycans in total glycans	SUM(GP18,GP32:33)	SUM(GP18,GP20,GP26,GP39,GP49,GP50/2,GP51)	SUM(GP20,GP33,GP45,GP47)	SUM(GP9, GP20:22,GP26,GP32,GP39,GP50/2,GP51)
	S2	The percentage of di-sialylated glycans in total glycans	SUM(GP27,GP30,GP39)	SUM(GP30,GP32,GP35,GP38,GP48)	SUM(GP30,GP38,GP42:43,GP46,GP49,GP56)	SUM(GP27,GP30,GP35,GP42,GP47,GP48/2,GP52,GP56)
	S3	The percentage of tri-sialylated glycans in total glycans	SUM(GP43:44,GP49, GP53,GP56, GP58)	SUM(GP43,GP46,GP50/2,GP53,GP56,GP58)	SUM(GP44,GP53)	SUM(GP43,GP46,GP48/2,GP49,GP53)
	S4		SUM(GP50,GP52,GP54,GP57)	SUM(GP47,GP52,GP54,GP57,GP59)	SUM(GP50,GP52,GP54,GP57:59)	SUM(GP50/2,GP54,GP57,GP59)
	Phos	The percentage of phosphorylated glycans in total glycans	ND	ND	ND	ND
	S^{Ac}	The percentage of acetylated charged species	ND	ND	ND	SUM(GP9)
	Total charged species	The percentage of total charged species in total glycans	SUM(GP18,GP27,GP30,GP32:33,GP39,GP43:44,GP49:50,GP52:54,GP56:59)	SUM(GP18,GP20,GP26,GP30,GP32,GP35,GP38,:39,GP43,GP46:54,GP56:59)	SUM(GP20,GP30,GP33,GP38,GP42:47,GP49:50,GP52:54,GP56:59)	SUM(GP9,GP20:22,GP26:27,GP30,GP32,GP35,GP39,GP42:43,GP46:54,GP56:59)
	Poly	The percentage of polysialic acids in total glycans	SUM(GP39,GP44,GP57,GP59)	SUM(GP32,GP46:47,GP50/2,GP57,GP59)	SUM(GP42,GP44,GP57:58)	SUM(GP43,GP46,GP47,GP57,GP58)
Branching	A1	The percentage of monoantennary glycans in total glycans	SUM(GP4)	SUM(GP4)	SUM(GP4)	SUM(GP4)
	A1B	The percentage of bisecting monoantennary glycans in total glycans	ND	ND	ND	ND
	A2	The percentage of biantennary glycans in total glycans	SUM(GP18,GP30,GP33,GP57)	SUM(GP6,GP20,GP30, GP48,GP57)	SUM(GP13,GP20,GP28,GP30,GP5	SUM(GP6/2,GP9,GP12,GP14,GP16/2,GP20,GP22,GP30,GP48/2,GP57)
	A2B	The percentage of bisecting diantennary glycans in total glycans	SUM(GP5,GP15, GP26)	SUM(GP13,GP18)	SUM(GP5,GP15/2,GP26)	SUM(GP15/2)
	A3	The percentage of triantennary glycans in total glycans	SUM(GP6, GP7/2,GP13,GP19:20, GP32,GP43:44,GP49)	SUM(GP7/2,GP26,GP28,GP38,GP43,GP47,GP49)	SUM(GP6,GP7/2, GP15/2,GP16,GP19,GP31,GP34,GP38,GP42,GP44)	SUM(GP6/2,GP7/2,GP15/2,GP21,GP26,GP32,GP38,GP43, GP49)
	A3B	The percentage of bisecting triantennary glycans in total glycans	SUM(GP22,GP25,GP27,GP35,GP39)	SUM(GP14,GP22,GP25/2,GP35,GP46)	SUM(GP21:22, GP49)	SUM(GP27,GP46)

			Healthy/ILBD		PD	
			Striatum	Substantia Nigra	Striatum	Substantia Nigra
**Branching**	A4	The percentage of tetraantennary glycans in total glycans	SUM(GP21,GP31,GP34,GP36:37,GP41,GP45:46,GP48,GP50:52,GP54,GP56,GP59)	SUM(GP10,GP19,GP25/2,GP27,GP31:34,GP36:37,GP41,GP44:45,GP51:52,GP54:56,GP59)	SUM(GP10,GP25,GP27,GP35:37,GP39,GP41,GP43,GP45,GP48,GP50,GP52,GP54,GP56,GP58)	SUM(GP10,GP13,GP19,GP24,GP28,GP31,GP34:37,GP41,GP44:45,GP47,GP48/2,GP50/2,GP51:52,GP54:56,GP58:59)
	A4B	The percentage of bisecting tetraantennary glycans in total glycans	SUM(GP40,GP47,GP53,GP55,GP58)	SUM(GP9,GP39,GP40,GP50/2,GP53,GP58)	SUM(GP9,GP33,GP40,GP46:47,GP51,GP53,GP55,GP59)	SUM(GP33,GP39:40,GP50/2,GP53)
**Bisects**	A1B, A2B, A3B, A4B	The percentage of bisecting glycans in total glycans	SUM(GP5,GP15,GP22,GP25:27,GP35,GP39:40,GP47,GP53,GP55,GP58)	SUM(GP8:9,GP13,GP14,GP22,GP25/2,GP35,GP39:40,GP46,GP50/2,GP53,GP58)	SUM(GP5,GP8:9,GP15/2,GP21:22,GP26,GP33,GP40,GP46,GP47,GP49,GP51,GP53,GP55,GP59)	SUM(GP15/2,GP27,GP46,GP33,GP39-40,GP50/2,GP53)
**Oligo-mannose**	Total oligo-mannose	The percentage of oligomannose structures in total glycans	SUM(GP1:3,GP7/4, GP8, GP11, GP14/2, GP16:17, GP23:24, GP29, GP38, GP42)	SUM(GP1:3,GP7/4,GP11,GP17,GP21,GP23:24,GP29,GP42)	SUM(1:3,GP8,GP11:12,GP14,GP17,GP23:24,GP29,GP7/4)	SUM(GP1:3,GP7/4,GP8,GP11,GP16/2,GP17:18,GP23,GP25,GP29)
	Lower order oligo-mannose	The percentage of oligomannose structures from M1-M5 in total glycans	SUM(GP1:3, GP7/5, GP8)	SUM(GP1:3,GP7/4)	SUM(1:3,GP8,GP7/4,GP12)	SUM(GP1:3,GP7/4,GP8)
	Higher order oligo-mannose	The percentage of oligomannose structures from M6-M9 in total glycans	SUM(GP11, GP14/2, GP16:17, GP23:24, GP29, GP38,GP42)	SUM(GP11,GP17,GP21,GP23:24,GP29,GP42)	SUM(GP11,GP14,GP17,GP23:24,GP29)	SUM(GP11,GP16/2,GP17:18,GP23,GP25,GP29,GP42)
**Hybrid**	Total hybrid glycans	The percentage of hybrid structures in total glycans	SUM(GP9, GP12)	SUM(GP5,GP8,GP12,GP15)	SUM(GP8,GP12)	SUM(GP5)
**Core fucosy-lation**	Core F^{Total}	The percentage of core fucosylated glycans in total glycans	SUM(GP2,GP4,GP7/2,GP8, GP10,GP15,GP18,GP21,GP25:26,GP31:36, GP39:41,GP44:46,GP48,GP51,GP52,GP55:56,GP58:59)	SUM(GP2,GP4,GP6,GP7/2,GP8,GP10,GP13,GP15,GP18:19,GP21,GP25/2,GP26:28,GP32,GP34,GP36,GP38,GP40:41,GP45:48,GP52,GP55:56,GP58)	SUM(GP2,GP4,GP7/2,GP8,GP10,GP13,GP15:16,GP18,GP21,GP25:28,GP34:36,GP38:41,GP43:45,GP48:49,GP52,GP55:56,GP58)	SUM(GP2,GP4,GP6/2,GP7/2,GP8,GP10,GP13:15,GP16/2,GP19,GP22,GP24,GP26,GP30,GP32,GP34:36,GP38,GP40:42,GP45:48,GP50/2,GP55:56,GP58)
**Outer arm fucosy-lation**	Outer arm F^{Total}	The percentage of outer arm fucosylated glycans in total glycans	SUM(GP14/2,GP15,GP18:19,GP26:27,GP33:34,GP36,GP40,GP44,GP46:49,GP55,GP58:59)	SUM(GP19,GP25/2,GP26:27,GP32:34,GP36,GP39:40,GP43:44,GP46:47,GP49:51,GP54,GP56,GP58)	SUM(GP13,GP15:16,GP19,GP21,GP25:28,GP31,GP36,GP39:40,GP42:45,GP48,GP51,GP54:55,GP58:59)	SUM(GP12,GP15,GP16/2,GP19,GP21,GP24,GP26,GP27,GP34,GP36,GP38:41,GP43,GP44,GP46,GP47,GP48/2,GP49,GP50/2,GP51,GP54,GP58)
**N-acetyl-galacto-samine**	GalNAc	The percentage of N-acetylgalactosimined structures in total glycans	ND	SUM(GP19,GP32,GP59)	SUM(GP18,GP28,GP31,GP39,GP51,GP58)	SUM(GP9,GP12:13,GP16/2,GP19,GP41,GP52,GP59)
**Lac**	Total Lac structures	The percentage of structures with Lac in total glycans	SUM(GP21,GP28,GP31,GP35,GP45,GP51,GP56)	SUM(GP45,GP55)	SUM(GP34,GP47,GP56)	SUM(GP28,GP45,GP55:56)

Table S2. Human brain tissue of Parkinson's disease (PD), incidental Lewy Bodies disease (ILBD) and control cases used in this study. Cntrl: control; PMD: post-mortem delay; N/A: not applicable

Case number	Gender	PMD (hours)	Age at death (years)	Duration of the disease (years)	Braak stage	Brain Bank identifier number
**DISEASE**						
PD1	M	21	79	12	3	PD014
PD2	F	14	76	10	4	PD022
PD3	F	10	80	13	4	PD063
PD4	F	22	87	9	4	PD086
PD5	M	9	72	6	4	PD109
PD6	F	5	86	18	3	PD204
PD7	M	11	72	2	3	PD572
PD8	F	19	80	13	3	PD576
PD9	M	9	76	21	4	PD579
PD10	F	16	82	11	3	PD590
PD11	M	17	77	9	4	PD591
PD12	M	16	85	15	4	PD596
PD13	M	11	78	16	4	PD612
PD14	F	24	83	12	4	PD683
PD15	M	16	86	19	3	PD687
PD16	F	5	88	5	3	PD709
PD17	F	17	87	13	4	PD712
ILBD1	F	10	104	N/A	ILBD	PDC007
ILBD2	F	28	77	N/A	ILBD	PDC082
ILBD3	F	39	82	N/A	ILBD	PDC108
**CONTROLS**						
Ctrl1	F	17	71	N/A	N/A	PDC008
Ctrl2	F	15	81	N/A	N/A	PDC015

Case number	Gender	PMD (hours)	Age at death (years)	Duration of the disease (years)	Braak stage	Brain Bank identifier number
Ctrl3	M	12	65	N/A	N/A	PDC022
Ctrl4	F	23	80	N/A	N/A	PDC026
Ctrl5	M	12	90	N/A	N/A	PDC034
Ctrl6	F	15	61	N/A	N/A	PDC040
Ctrl7	F	22	89	N/A	N/A	PDC053
Ctrl8	F	20	82	N/A	N/A	PDC085
Ctrl9	F	24	96	N/A	N/A	PDC088
Ctrl10	M	11	95	N/A	N/A	PDC105
Ctrl11	F	15	87	N/A	N/A	PDC107
Ctrl12	F	23	88	N/A	N/A	PDC111
Ctrl13	M	22	77	N/A	N/A	C045
Ctrl14	M	10	68	N/A	N/A	C048
Ctrl15	M	16	66	N/A	N/A	C054
Ctrl16	F	21	63	N/A	N/A	C064
Ctrl17	F	22	84	N/A	N/A	C074
Ctrl18	M	8	88	N/A	N/A	C075
Ctrl19	F	24	87	N/A	N/A	C083
Ctrl20	F	23	84	N/A	N/A	C084
Ctrl21	F	22	81	N/A	N/A	C085
Ctrl22	F	18	70	11	N/A	PD529

Table S3. Lectins used for the microarray, binding specificity and inhibitory carbohydrate

Lectin	Abbreviation	Specificity	Inhibitory carbohydrate
Concanavalin A	ConA	α-Man, α-Glc	Mannose
Narcissus Pseudonarcissus	NPL	Terminal and internal Man	Mannose
Vicia faba lectin	VFA	α-Man, Glc, GlcNAc	Mannose
Allium sativum lectin	ASA	α-1,3Man	Mannose
Galanthus nivalin agglutinin	GNA	α-D-Man, terminal α-1,3Man	Mannose
Triticum Vulgaris	WGA	(GlcNAc)n, Sialic acid	GlcNAc
Phytolacca americana	PWA	(GlcNAc)3	GlcNAc
Griffonia simplicifolia II	BS-II	Terminal GlcNAc	GlcNAc
Lycopersicon esculentum	LEL	(GlcNAc)3	GlcNAc
Solanum tuberosum	STL	(GlcNAc)3, LacNAc	GlcNAc
Pseudomonas aeruginosa PA-I	PAL	Galactose	Galactose
Datura stramonium	DSL	(β-1,4) linked *N*-Acetylglucosamine oligomers, (GlcNAc)2-3, LacNAc	GlcNAc
Ricinum communis agglutinin	RCA_{120}	β -Gal, Lac, LacNAc	Galactose
Erythrina cristagalli A	ECA	Gal, GalNAc	GalNAc
Phaseolus vulgaris agglutinin	PHA E+L	Oligosaccharides	Lactose
Griffonia simplicifolia I	BS-I-B4	α-Gal	Galactose
Cicer arietinum	CAL	Fetuin, Lac, IgM	Lactose
Maackia amurensis Lectin II	MAL-II	LacNAc	Galactose
Sambucus nigra	SNA	Sialic acid-α-2,6–GalNAc	Lactose, Sialic acid
Maackia amurensis Lectin I	MAL-I	Sialic acid-α-2,3–GalNAc	Lactose, Sialic acid
Homarus americanus	HMA	LAg1 NeuNAc. LAg2: GalNAc	GalNAc
Ulex Europaea Agglutinin	UEA-I	α-1,2-fucose	Fucose
Pisum sativum	PSA	Fucose -α-1,6-GlcNAc, α-Man	Fucose
Lotus tetragonolobus	LTL	Terminal α-Fucose, Le^x	Fucose
Aspergillus orzyae	AOL	Fucose	Fucose
Aleuria aurantia lectin	AAL	α –1,6-Fuc to *N*-Acetylglucosamine, α-1,3-Fuc to *N*-Acetyllactosamine	Fucose

Anguilla anguilla agglutinin	AAA	D-Fucose, α-1,2-Fucose, α-1,4-Fucose	Fucose
Vicia villosa B4	VVL	GalNAc	GalNAc
Jacalin	JAC	T antigen	GalNAc
Agaricus bisporus	ABL	Gal- β 1,3-GalNAc, T antigen	Galactose
Amarantus Caudatus	ACA	T antigen, (Gal-β 1,3-GalNAc α -Thr/Ser)	Galactose
Maclura Pomifera	MPA	T antigen, αGalNAc	Galactose
Psophocarpus tetragonolobus II	PT-II	α-1,2-fucosylated LacNAc	Galactose
Griffonia simplicifolia I	BS-I	α-Gal	Galactose
Euonymus Europaeus	EEA	Lac, blood groups B and H	Galactose
Marasmium oreades agglutinin	MOA	Gal-α1,3Gal and Gal-α1,3Gal-β1,4GlcNAc	Galactose
Helix pomatia	HPL	α-GalNAc terminal	GalNAc
Sophora japonica	SJA	GalNAc	GalNAc
Helix aspersa lectin	HAL	α-GalNAc terminal	GalNAc
Soybean agglutinin	SBA	α-Gal-GalNAc	GalNAc
Peanut agglutinin	PNA	T antigen, Gal β-1,3-GalNAc	Galactose
Dolichos biflorus agglutinin	DBA	Terminal GalNAc	GalNAc
Wisteria floribunda	WFL	GalNAc	GalNAc
Salvia sclarea lectin	SSA	Terminal GalNAc linked to serine	GalNAc
Psophocarpus tetragonolobus I	PT-I	α-GalNAc	GalNAc
Bauhinia Purpurea lectin	BPL	GalNAc	GalNAc

Table S4. Lectins used for cytochemistry, their binding specificity, concentration used and inhibitory carbohydrate (haptenic sugar, 150 mM).

Lectin	Abbreviation	Binding specificity	Concentration	Inhibitory carbohydrate
Sambucus nigra agglutinin isolectin-I	SNA-I	Neu5Ac/Gc-α(2,6)-Gal/GalNAc-R	20 µg/mL	Lactose
Anguilla anguilla agglutinin	AAA	D-Fucose, α-1,2-Fucose, α-1,4-Fucose	10 µg/mL	Fucose
Datura stramonium	DSL	(β-1,4) linked N-acetylglucosamine oligomers, (GlcNAc)2-3, LacNAc	15 µg/mL	GlcNAc, GalNAc
Ricinus communis Agglutinin	RCA	β -Gal, Lac, LacNAc	5 µg/mL	Gal or lactose
Galanthus nivalin agglutinin	GNA	α-D-Man, terminal α-1,3Man	10 µg/mL	Mannose

Table S5. Genes present in the PCR RT2- Profiler Human Glycosylation Array (Qiagen, UK)

RefSeq Number	Description	Symbol
NM_016161	Alpha-1,4-N-acetylglucosaminyltransferase	A4GNT
NM_000027	Aspartylglucosaminidase	AGA
NM_194318	Beta 1,3-galactosyltransferase-like	B3GLCT
NM_006577	UDP-GlcNAc:betaGal beta-1,3-N-acetylglucosaminyltransferase 2	B3GNT2
NM_014256	UDP-GlcNAc:betaGal beta-1,3-N-acetylglucosaminyltransferase 3	B3GNT3
NM_030765	UDP-GlcNAc:betaGal beta-1,3-N-acetylglucosaminyltransferase 4	B3GNT4
NM_198540	UDP-GlcNAc:betaGal beta-1,3-N-acetylglucosaminyltransferase 8	B3GNT8
NM_001497	UDP-Gal:betaGlcNAc beta 1,4- galactosyltransferase, polypeptide 1	B4GALT1
NM_003780	UDP-Gal:betaGlcNAc beta 1,4- galactosyltransferase, polypeptide 2	B4GALT2
NM_003779	UDP-Gal:betaGlcNAc beta 1,4- galactosyltransferase, polypeptide 3	B4GALT3
NM_004776	UDP-Gal:betaGlcNAc beta 1,4- galactosyltransferase, polypeptide 5	B4GALT5
NM_020156	Core 1 synthase, glycoprotein-N-acetylgalactosamine 3-beta-galactosyltransferase, 1	C1GALT1
NM_152692	C1GALT1-specific chaperone 1	C1GALT1C1
NM_014674	ER degradation enhancer, mannosidase alpha-like 1	EDEM1
NM_018217	ER degradation enhancer, mannosidase alpha-like 2	EDEM2
NM_025191	ER degradation enhancer, mannosidase alpha-like 3	EDEM3
NM_000147	Fucosidase, alpha-L- 1, tissue	FUCA1
NM_032020	Fucosidase, alpha-L- 2, plasma	FUCA2
NM_173540	Fucosyltransferase 11 (alpha (1,3) fucosyltransferase)	FUT11
NM_178157	Fucosyltransferase 8 (alpha (1,6) fucosyltransferase)	FUT8
NM_020474	UDP-N-acetyl-alpha-D-galactosamine:polypeptide N-acetylgalactosaminyltransferase 1 (GalNAc-T1)	GALNT1
NM_198321	UDP-N-acetyl-alpha-D-galactosamine:polypeptide N-acetylgalactosaminyltransferase 10 (GalNAc-T10)	GALNT10
NM_022087	UDP-N-acetyl-alpha-D-galactosamine:polypeptide N-acetylgalactosaminyltransferase 11 (GalNAc-T11)	GALNT11
NM_024642	UDP-N-acetyl-alpha-D-galactosamine:polypeptide N-acetylgalactosaminyltransferase 12 (GalNAc-T12)	GALNT12
NM_052917	UDP-N-acetyl-alpha-D-galactosamine:polypeptide N-acetylgalactosaminyltransferase 13 (GalNAc-T13)	GALNT13

RefSeq Number	Description	Symbol
NM_024572	UDP-N-acetyl-alpha-D-galactosamine:polypeptide N-acetylgalactosaminyltransferase 14 (GalNAc-T14)	GALNT14
NM_004481	UDP-N-acetyl-alpha-D-galactosamine:polypeptide N-acetylgalactosaminyltransferase 2 (GalNAc-T2)	GALNT2
NM_004482	UDP-N-acetyl-alpha-D-galactosamine:polypeptide N-acetylgalactosaminyltransferase 3 (GalNAc-T3)	GALNT3
NM_003774	UDP-N-acetyl-alpha-D-galactosamine:polypeptide N-acetylgalactosaminyltransferase 4 (GalNAc-T4)	GALNT4
NM_007210	UDP-N-acetyl-alpha-D-galactosamine:polypeptide N-acetylgalactosaminyltransferase 6 (GalNAc-T6)	GALNT6
NM_017423	UDP-N-acetyl-alpha-D-galactosamine:polypeptide N-acetylgalactosaminyltransferase 7 (GalNAc-T7)	GALNT7
NM_017417	UDP-N-acetyl-alpha-D-galactosamine:polypeptide N-acetylgalactosaminyltransferase 8 (GalNAc-T8)	GALNT8
NM_021808	UDP-N-acetyl-alpha-D-galactosamine:polypeptide N-acetylgalactosaminyltransferase 9 (GalNAc-T9)	GALNT9
NM_020692	UDP-N-acetyl-alpha-D-galactosamine:polypeptide N-acetylgalactosaminyltransferase-like 1	GALNT16
NM_145292	UDP-N-acetyl-alpha-D-galactosamine:polypeptide N-acetylgalactosaminyltransferase-like 5	GALNTL5
NM_001034845	UDP-N-acetyl-alpha-D-galactosamine:polypeptide N-acetylgalactosaminyltransferase-like 6	GALNTL6
NM_198334	Glucosidase, alpha; neutral AB	GANAB
NM_001490	Glucosaminyl (N-acetyl) transferase 1, core 2	GCNT1
NM_004751	Glucosaminyl (N-acetyl) transferase 3, mucin type	GCNT3
NM_016591	Glucosaminyl (N-acetyl) transferase 4, core 2	GCNT4
NM_000404	Galactosidase, beta 1	GLB1
NM_024312	N-acetylglucosamine-1-phosphate transferase, alpha and beta subunits	GNPTAB
NM_032520	N-acetylglucosamine-1-phosphate transferase, gamma subunit	GNPTG
NM_000520	Hexosaminidase A (alpha polypeptide)	HEXA
NM_000521	Hexosaminidase B (beta polypeptide)	HEXB
NM_005907	Mannosidase, alpha, class 1A, member 1	MAN1A1
NM_006699	Mannosidase, alpha, class 1A, member 2	MAN1A2
NM_016219	Mannosidase, alpha, class 1B, member 1	MAN1B1
NM_020379	Mannosidase, alpha, class 1C, member 1	MAN1C1

RefSeq Number	Description	Symbol
NM_002372	Mannosidase, alpha, class 2A, member 1	MAN2A1
NM_006122	Mannosidase, alpha, class 2A, member 2	MAN2A2
NM_000528	Mannosidase, alpha, class 2B, member 1	MAN2B1
NM_005908	Mannosidase, beta A, lysosomal	MANBA
NM_002406	Mannosyl (alpha-1,3-)-glycoprotein beta-1,2-N-acetylglucosaminyltransferase	MGAT1
NM_002408	Mannosyl (alpha-1,6-)-glycoprotein beta-1,2-N-acetylglucosaminyltransferase	MGAT2
NM_002409	Mannosyl (beta-1,4-)-glycoprotein beta-1,4-N-acetylglucosaminyltransferase	MGAT3
NM_012214	Mannosyl (alpha-1,3-)-glycoprotein beta-1,4-N-acetylglucosaminyltransferase, isozyme A	MGAT4A
NM_014275	Mannosyl (alpha-1,3-)-glycoprotein beta-1,4-N-acetylglucosaminyltransferase, isozyme B	MGAT4B
NM_013244	Mannosyl (alpha-1,3-)-glycoprotein beta-1,4-N-acetylglucosaminyltransferase, isozyme C (putative)	MGAT4C
NM_002410	Mannosyl (alpha-1,6-)-glycoprotein beta-1,6-N-acetyl-glucosaminyltransferase	MGAT5
NM_144677	Mannosyl (alpha-1,6-)-glycoprotein beta-1,6-N-acetyl-glucosaminyltransferase, isozyme B	MGAT5B
NM_006302	Mannosyl-oligosaccharide glucosidase	MOGS
NM_016256	N-acetylglucosamine-1-phosphodiester alpha-N-acetylglucosaminidase	NAGPA
NM_000434	Sialidase 1 (lysosomal sialidase)	NEU1
NM_005383	Sialidase 2 (cytosolic sialidase)	NEU2
NM_006656	Sialidase 3 (membrane sialidase)	NEU3
NM_080741	Sialidase 4	NEU4
NM_181673	O-linked N-acetylglucosamine (GlcNAc) transferase (UDP-N-acetylglucosamine:polypeptide-N-acetylglucosaminyl transferase)	OGT
NM_172236	Protein O-fucosyltransferase 1	POFUT1
NM_133635	Protein O-fucosyltransferase 2	POFUT2
NM_017739	Protein O-linked mannose beta1,2-N-acetylglucosaminyltransferase	POMGNT1
NM_007171	Protein-O-mannosyltransferase 1	POMT1
NM_013382	Protein-O-mannosyltransferase 2	POMT2
NM_002743	Protein kinase C substrate 80K-H	PRKCSH

RefSeq Number	Description	Symbol

NM_173344	ST3 beta-galactoside alpha-2,3-sialyltransferase 1	ST3GAL1
NM_006927	ST3 beta-galactoside alpha-2,3-sialyltransferase 2	ST3GAL2
NM_003032	ST6 beta-galactosamide alpha-2,6-sialyltranferase 1	ST6GAL1
NM_018414	ST6 (alpha-N-acetyl-neuraminyl-2,3-beta-galactosyl-1,3)-N-acetylgalactosaminide alpha-2,6-sialyltransferase 1	ST6GALNAC1
NM_006011	ST8 alpha-N-acetyl-neuraminide alpha-2,8-sialyltransferase 2	ST8SIA2
NM_015879	ST8 alpha-N-acetyl-neuraminide alpha-2,8-sialyltransferase 3	ST8SIA3
NM_175052	ST8 alpha-N-acetyl-neuraminide alpha-2,8-sialyltransferase 4	ST8SIA4
NM_001004470	ST8 alpha-N-acetyl-neuraminide alpha-2,8-sialyltransferase 6	ST8SIA6
NM_020120	UDP-glucose glycoprotein glucosyltransferase 1	UGGT1
NM_020121	UDP-glucose glycoprotein glucosyltransferase 2	UGGT2
NM_001101	Actin, beta	ACTB
NM_004048	Beta-2-microglobulin	B2M
NM_002046	Glyceraldehyde-3-phosphate dehydrogenase	GAPDH
NM_000194	Hypoxanthine phosphoribosyltransferase 1	HPRT1
NM_001002	Ribosomal protein, large, P0	RPLP0

Table S6. List of primers used for RT-PCR.

Gene/Primer	Forward sequence	Reverse sequence
18S (housekeeping)	AATCAGTTATGGTTCCTTTGTCG	GCTCTAGAATTACCACAGTTATCCAA
ATF6	GGACCAGGTGGTGTCAGAG	GACAGCTCTGCGCTTTGG
CHOP	GAAATCGAGCGCCTGACCAG	GGAGGTGATGCCAACAGTTCA
XBP1 unspliced	CAGACTACGTGCGCCTCTG	CTTCTGGGTAGACCTCTGGG
XBP1 spliced	GAGTCCGCAGCAGGTGC	GGTCCAACTTGTCCAGAATGC

Table S7 (Datasheet). Exoglycosidase digestion panels for healthy striatum pool (Tables S7.1/S7.2), PD striatum pool (Tables S7.3/S7.4), healthy substantia nigra pool (Tables S7.5/S7.6), PD substantia nigra pool (Tables S7.7/S7.8). – REFER TO SUPPLEMENTARY SPREADSHEET FILE.

Table S8 (Datasheet). HILIC-UPLC-MS data from healthy striatum pool (Table S8.1), PD striatum pool (Table S8.2), healthy substantia nigra pool (Table S8.3), PD substantia nigra pool (Table S8.4), outlining m/z values as well as elution time (min), relative charge state observed, error (ppm) and monosaccharide composition. - REFER TO SUPPLEMENTARY SPREADSHEET FILE.

SI References

(1) Kooner A.S., et al, Synthesis of N-Glycolylneuraminic Acid (Neu5Gc) and Its Glycosides, *Front. Immunol.* 2019. 10, 2004.

(2) Akasaka-manya K., et al, Protective effect of N-glycan bisecting GlcNAc residues on β-amyloid production in Alzheimer's disease, *Glycobiology.* 2010. 20, 99–106

(3) Royle L., et al, Detailed structural analysis of N-glycans released from glycoproteins in SDS-PAGE gel bands using HPLC combined with exoglycosidase array digestions, *Methods Mol. Biol.* 2006. 347, 125–143.

(4) Angel P.M., et al. MALDI Imaging Mass Spectrometry of N-glycans and Tryptic Peptides from the Same Formalin-Fixed, Paraffin-Embedded Tissue Section, Methods Mol. Biol. 2018. 1788, 225–241.
